# Type 2 diabetes detection and management among insured adults

**DOI:** 10.1186/s12963-016-0110-4

**Published:** 2016-11-21

**Authors:** Timothy M. Dall, Weyna Yang, Pragna Halder, Jerry Franz, Erin Byrne, April P. Semilla, Ritashree Chakrabarti, Bruce Stuart

**Affiliations:** 1 IHS Markit, 1150 Connecticut Ave., NW, Suite 401, Washington, DC 20036 USA; 2 The Lewin Group, 3130 Fairview Park Drive, Suite 500, Falls Church, VA 22042 USA; 3 Novo Nordisk Inc., 800 Scudders Mill Road, Plainsboro, NJ 08536 USA; 4 The Peter Lamy Center on Drug Therapy and Aging, Department of Pharmaceutical Health Services Research, University of Maryland Baltimore, 220 Arch St., Room 12-212, Baltimore, MD 21201 USA

**Keywords:** Diabetes, Detection, Management

## Abstract

**Background:**

The Centers for Disease Control and Prevention estimates that 28.9 million adults had diabetes in 2012 in the US, though many patients are undiagnosed or not managing their condition. This study provides US national and state estimates of insured adults with type 2 diabetes who are diagnosed, receiving exams and medication, managing glycemic levels, with diabetes complications, and their health expenditures. Such information can be used for benchmarking and to identify gaps in diabetes detection and management.

**Methods:**

The study combines analysis of survey data with medical claims analysis for the commercially insured, Medicare, and Medicaid populations to estimate the number of adults with diagnosed type 2 diabetes and undiagnosed diabetes by insurance type, age, and sex. Medical claims analysis used the 2012 de-identified Normative Health Information database covering a nationally representative commercially insured population, the 2011 Medicare 5% Sample, and the 2008 Medicaid Mini-Max.

**Results:**

Among insured adults in 2012, approximately 16.9 million had diagnosed type 2 diabetes, 1.45 million had diagnosed type 1 diabetes, and 6.9 million had undiagnosed diabetes. Of those with diagnosed type 2, approximately 13.0 million (77%) received diabetes medication-ranging from 70% in New Jersey to 82% in Utah. Suboptimal percentages had claims indicating recommended exams were performed. Of those receiving diabetes medication, 43% (5.6 million) had medical claims indicating poorly controlled diabetes-ranging from 29% with poor control in Minnesota and Iowa to 53% in Texas. Poor control was correlated with higher prevalence of neurological complications (+14%), renal complications (+14%), and peripheral vascular disease (+11%). Patients with poor control averaged $4,860 higher average annual health care expenditures-ranging from $6,680 for commercially insured patients to $4,360 for Medicaid and $3,430 for Medicare patients.

**Conclusions:**

This study highlights the large number of insured adults with undiagnosed type 2 diabetes by insurance type and state. Furthermore, this study sheds light on other gaps in diabetes care quality among patients with diagnosed diabetes and corresponding poorly controlled diabetes. These findings underscore the need for improvements in data collection and diabetes screening and management, along with policies that support these improvements.

**Electronic supplementary material:**

The online version of this article (doi:10.1186/s12963-016-0110-4) contains supplementary material, which is available to authorized users.

## Background

Diabetes is a complex disease that if poorly controlled increases risk for cardiovascular disease, neuropathy, retinopathy, nephropathy, and other medical conditions [1]. The Centers for Disease Control and Prevention (CDC) estimates that in 2012 the US had 28.9 million adults (age 20 or older) with diabetes, including 20.8 million with diagnosed diabetes and 8.1 million whose diabetes was undiagnosed [2]. Among adults with diagnosed diabetes (type 1 and type 2), self-reported data captured through national surveys suggest 86% (17.9 million) used oral medication or insulin to control glycemic levels, 71% used medication for hypertension, and 65% used medication for hypercholesterolemia [2]. Diabetes is a costly disease, and in 2012 was estimated to have contributed to $176 billion in excess medical costs [3, 4]. Type 2 diabetes (T2DM) accounts for 90 to 95% of diabetes cases, and both T2DM and its complications are potentially preventable [5]. National statistics suggest a substantial proportion of cases are undiagnosed, untreated, not under optimal control, and at high risk for complications and associated medical and indirect costs [6–8].

This study combined survey and medical claims analysis to address key research questions by state and insurance type: (1) How many insured adults in 2012 had diagnosed and undiagnosed T2DM? (2) Among diagnosed T2DM cases, how many received diabetes medication and recommended exams? (3) Among patients who received diabetes medication, how many had poorly controlled diabetes as indicated by ICD-9 diagnosis codes? (4) How did the prevalence of diabetes complications and annual medical expenditures differ by diabetes control status? This information provides benchmarks to track improvements in diabetes detection and management as more people in each state gain insurance coverage under the Affordable Care Act, as new diabetes screening recommendations are implemented [9], and as integrated health care systems seek better ways to improve detection and management.

## Methods

The breadth of this study required multiple data sources and methods, with data covering several years sometimes combined to increase sample size. Data prior to 2012 were used when 2012 data were unavailable. Estimates of diabetes prevalence and diagnosed status by state, age, sex, and insurance type came from national surveys. Analysis of medical claims provided information on medical expenditures and the proportion of diabetes cases that were T2DM, were receiving medication and recommended exams, had indications of poorly controlled diabetes, and had diabetes-related complications. This study used secondary data sources and received an exemption from the New England Institutional Review Board.

### Estimating prevalence of diagnosed and undiagnosed diabetes

The approach to estimate prevalence of diagnosed and undiagnosed diabetes by population age (20–34, 35–44, 45–54, 55–59, 60–64, 65–70, and over 70), sex, insurance type (commercial, Medicare, Medicaid, and uninsured), and state is documented elsewhere, though detailed prevalence estimates by insurance type have not been previously published [3, 4, 10]. Using the 2012 American Community Survey (ACS, *n* = 2,375,715), we constructed a population file where each person residing in the community was matched with a similar person from the combined 2011 and 2012 Behavioral Risk Factor Surveillance System (BRFSS, *n* = 982,154) and each person residing in a nursing home was matched with a similar person from the 2004 National Nursing Home Survey (NNHS, *n* = 14,017). Detailed information on file construction is published elsewhere, but the ACS-BRFSS match criteria used state, age, sex, race/ethnicity, medical insurance status, and household income [3, 4]. The ACS-NNHS match criteria used age, sex, and race/ethnicity. The 2011 and 2012 BRFSS files were combined to increase sample size and assume little change in diabetes diagnosis rates between 2011 and 2012 controlling for patient demographics. The 2004 NNHS is the most recently available person-level file for the nursing home population, but the underlying rate of diagnosed diabetes was adjusted to reflect a 2011 national study that found diabetes prevalence was close to 33% among nursing home residents [11].

Diagnosed diabetes status (and hypertension and hyperlipidemia status) from BRFSS was determined based on previously having been told by a health professional that the respondent had the condition. Diabetes status from NNHS was determined by ICD-9 diagnosis code (250.xx); hypertension and hyperlipidemia status were determined by ICD 401.xx and 272.xx, respectively. Applying ACS sampling weights provides state-level estimates of adults with diagnosed diabetes and prevalence of conditions such as hypertension and hyperlipidemia among this population by demographic and insurance type.

To estimate prevalence of undiagnosed diabetes by state, age group, sex, and insurance type, we extrapolated national rates to states using the detailed state population characteristics available in the constructed ACS-BRFSS-NNHS file. We first analyzed national data in the combined 2009–2010 and 2011–2012 files of the National Health and Nutrition Examination Survey (NHANES) for adults who (a) had not previously been told by a health professional they had diabetes; (b) were not taking insulin; (c) were not pregnant; and (d) had laboratory results for hemoglobin A1c, fasting plasma glucose test, 2-h oral glucose tolerance test, or a combination of tests. We estimated a logistic regression where the dependent variable was diabetes (*n* = 649) as determined by laboratory results (see Additional file 1) exceeding thresholds for diabetes, versus no diabetes (*n* = 9,296). The explanatory variables were age group, sex, race/ethnicity, body weight category, insurance type, current smoker, year, and previous history or diagnosis of asthma, arthritis, heart attack, stroke, cancer, hypertension, hypercholesterolemia, and cardiovascular disease. We applied regression results (see Additional file 1) to the constructed ACS-BRFSS-NNHS 2012 population file to estimate the overall prevalence of undiagnosed diabetes by state.

### Estimating prevalence of T2DM among the insured population

We analyzed 2011–2012 medical and pharmacy claims for commercially insured adults in OptumInsight’s de-identified Normative Health Information (dNHI) database) (*n* = 29,948,496), for the Medicare population using the 2011 Medicare Standard Analytical File 5% sample (*n* = 2,805,812), and for the Medicaid population using the 2008 Mini-Max file (*n* = 3,095,634). All analyses were done by state, age, and sex. The dNHI database consists of longitudinally linked and de-identified individual-level data from one of the nation’s largest private insurance plans. The Medicare extract contains medical and prescription claims. Mini-Max is a 5% sample of the Medicaid Analytic eXtract data-a set of person-level data files on service utilization and payments for more than 60 million Medicaid enrollees extracted from the Medicaid Statistical Information System. Patients analyzed in each of these databases were continuously enrolled in a fee-for-service coverage type plan with no more than one gap in enrollment of up to 45 days during the measurement year for the commercially insured population, and were enrolled for all 12 months for the Medicare and Medicaid populations.

We identified patients with diabetes if the patient had at least one emergency department visit or hospitalization or two separate ambulatory visits with a diabetes diagnosis (ICD-9 of 250.xx) submitted during the year, or if the patient used insulin or other diabetes-related medications. Sample inclusion and exclusion criteria and the algorithm for distinguishing whether a patient had type 1 or type 2 diabetes are described in Additional file 1. Patients with a diagnosis of gestational diabetes were excluded. This analysis assumes that within strata defined by age group and sex, the proportion of diabetes cases that are T2DM remained relatively constant between 2012 and 2008 for the Medicaid population and between 2012 and 2011 for the Medicare population.

### Estimating the proportion of T2DM patients receiving medication and exams

Using pharmacy claims from the dNHI, Medicaid Mini-MAX data, and Medicare Part D files for each population strata, we calculated the percentage of patients with claims for insulin, non-insulin injectables, or oral antidiabetic agents. We calculated the percentage of patients with at least one claim for anti-hypertensive medications, statins, and angiotensin-converting enzyme (ACE) inhibitors/angiotensin II receptor blockers (ARB). Using procedure codes (see Additional file 1) we calculated the percentage of patients in each population stratum with indication of at least one cholesterol screening test, urine albumin test, or retinal eye exam during the year. These medications and exams are measures recommended by the HEDIS Comprehensive Diabetes Care 2012, and HEDIS is the source of the drug codes and procedure codes used.

### Estimating the characteristics and medical expenditures of treated T2DM patients by controlled status

Whether people have their diabetes under control is generally determined by hemoglobin A1c levels – with A1c < 7% often considered tight control, A1c > 9% considered uncontrolled, and recommended individual patient targets as high as 8.5% depending on a patient’s circumstances [1]. Lab results with A1c values were available only for a subset of the commercially insured patients and unavailable for Medicare and Medicaid beneficiaries. Therefore, to identify patients with uncontrolled diabetes, we used ICD-9 diagnosis codes of 250.x2 and 250.x3 in any claim during the year. For discussion purposes, we refer to “uncontrolled” or “poorly controlled” status for anyone with an ICD-9 code indicating uncontrolled diabetes at some point during the year, and use the term “controlled” to define patients with no indication of uncontrolled diabetes. Use of one claim for uncontrolled could overestimate the number of patients with uncontrolled diabetes; however, use of a diagnosis code rather than A1c results could also miss some patients with uncontrolled diabetes, so there are potentially errors in both directions. Later we discuss the limitations of using ICD-9 versus A1c to define uncontrolled status.

We used primary ICD-9 diagnosis codes (see Additional file 1) in medical claims to identify the presence of eight categories of diabetes complications: neurological symptoms, peripheral vascular disease, cardiovascular disease, renal complications, endocrine/metabolic complications, ophthalmic complications, other complications, and orthopedic problems. Using medical claims to indicate comorbidity presence could undercount prevalence of complications. Diabetes is one of multiple risk factors for these complications, and we report total medical expenditures by category because estimating the proportion of complications attributable to diabetes is beyond the scope of this study. We calculated the total annual medical expenditures and pharmacy expenditures for all medical conditions, with all costs inflated to 2012 dollars using the medical and pharmacy cost components of the Consumer Price Index.

## Results

### Diabetes prevalence

**Fig. 1:**
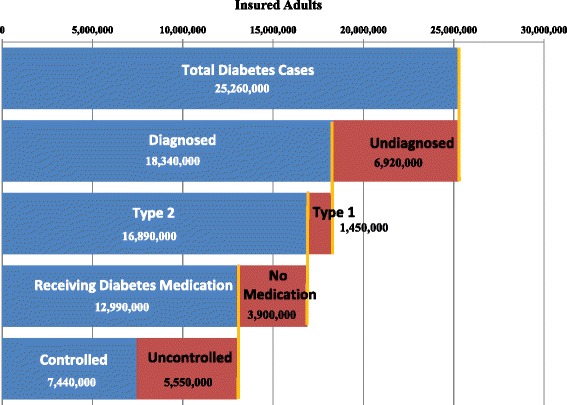
Insured adults with diabetes, 2012

We estimate that close to 25.3 million insured adults (age 20 or older) in the US had diabetes in 2012 (Fig. 1, Table 1). This includes over 18.3 million adults with diagnosed diabetes and 6.9 million undiagnosed. Of the insured, diagnosed population, approximately 16.9 million (about 92%) had T2DM, and 1.45 million had type 1 diabetes. The percent T2DM ranged from 87.1% for the commercially insured population to 97.6% for the Medicare population, and across the insured population ranged from approximately 88% in Utah to 95% in New Mexico (see Additional file 2 for all state-level estimates). The proportion of undiagnosed diabetes cases that are T2DM is unknown. Applying age-specific percent T2DM rates to the undiagnosed diabetes population suggests 6.3 million of the 6.9 million insured adults with undiagnosed diabetes probably have T2DM, though this is likely a lower bound for the proportion of T2DM.

**Table 1:** Diagnosed and undiagnosed adults with diabetes, 2012

National metrics	Insured				Uninsured
	Total	Commercial	Medicare^a^	Medicaid^b^	
Total with diabetes	25,260,000	11,400,000	11,430,000	2,430,000	4,950,000
Diagnosed	18,340,000	8,290,000	8,280,000	1,770,000	3,740,000
Percent diabetes cases type 2	92.1%	87.1%	97.6%	89.8%	82.0%^c^
Adults with diagnosed type 2 diabetes	16,890,000	7,220,000	8,080,000	1,590,000	3,440,000
Undiagnosed	6,920,000	3,110,000	3,150,000	660,000	1,210,000
Percent of type 2 diabetes patients undiagnosed^d^	27%	27%	28%	27%	23%

*Note*: ^a^Diabetes population estimates include only adults age 65 and older-including Medicaid/Medicare dual-eligible patients and veterans receiving care through the Veterans Health Administration

^b^Diabetes population estimates include only adults under age 65 who participate in a government-sponsored insurance program-including Medicaid/Medicare dual-eligible patients; veterans receiving care through the Veterans’ Health Administration; and beneficiaries enrolled in other federal or state-subsidized medical insurance plans (e.g., Indian Health Service)

^c^The percent type 2 was estimated using age-specific proportions of type 2 among the insured population

^d^Undiagnosed cases in each age group are assumed to have the same proportion type 2 as diagnosed cases in each age group

California (with 12% of the nation’s adult population) has an estimated 3.4 million people with diabetes (11% of the national total) including over 1 million with undiagnosed diabetes. Estimates of undiagnosed diabetes prevalence are based on the presence of risk factors among the population not diagnosed with diabetes. Alaska had the highest estimated proportion of diabetes cases that are undiagnosed, at 38%, whereas West Virginia (a state with high prevalence of diagnosed diabetes) had the lowest estimated proportion (20%) of cases undiagnosed.

Among those with diagnosed diabetes, 14% nationwide were without medical insurance in 2012 (pre Affordable Care Act), with this percentage ranging from a high of 21% in Louisiana to lows in the 5–6% range for Hawaii, Massachusetts, and Washington, DC.

### Exams and medication use

The medical claims analysis found that among the populations of insured adults diagnosed with T2DM, 82% had a medical claim for at least one A1c test during the year-ranging from 64% of the Medicaid population to 86% of the Medicare population (Table 2). Approximately 13% had at least one FPG test, and 76% had their cholesterol levels tested (ranging from 81% for Medicare to 58% for Medicaid). Only 57% of Medicare patients and 33% of commercially insured patients had claims indicating an eye exam during the year, with data unavailable for Medicaid patients. The calculated low rates for eye exams could reflect that some commercially insured patients potentially have a separate eye care plan or pay out-of-pocket, so not all eye exams are captured in claims data. Half (50%) of patients had claims for a urine albumin test. While not directly comparable, self-reported data from national surveys suggest 68.5% of all adults with diagnosed diabetes (type 1 and type 2, insured and uninsured) had two or more A1c tests in 2010; 62.8% had a dilated eye exam; and 85.6% took medication for diabetes (pills, insulin, or both) [2].

**Table 2:** Diabetes management of insured adults with diagnosed type 2 diabetes

Treatment metrics	Total insured	Commercial	Medicare	Medicaid
Annual glucose testing				
% receiving A1c	82%	80%	86%	64%
% receiving FPG	12%	10%	14%	15%
% receiving OGTT	0.6%	1.1%	0.2%	0.8%
Other recommended testing				
% receiving cholesterol test	76%	75%	81%	58%
% receiving eye exam	NA	33%	57%	NA
% receiving urinary albumin test	50%	45%	55%	43%
Medication to treat diabetes^a^				
% receiving diabetes medication	77%	82%	73%	75%
Receiving diabetes medication	12,990,000	5,930,000	5,860,000	1,200,000
Receiving metformin	46%	53%	40%	46%
Receiving insulin	23%	21%	23%	29%
Not receiving diabetes medication	3,900,000	1,290,000	2,220,000	390,000
Receiving other medications				
ACE inhibitors and ARBs^b^	63%	60%	67%	57%
Other anti-hypertensives (e.g., diuretics)	47%	31%	62%	43%
Statins	58%	55%	64%	46%
% with hypertension	80%	72%	91%	61%
% with hyperlipidemia	76%	75%	83%	43%

*Notes*: ^a^Includes insulin, non-insulin injectables, or oral antidiabetic agents

^b^ *ACE* Angiotensin-converting enzyme inhibitors, *ARB* Angiotensin II receptor blockers

Prescription claims for patients with diagnosed T2DM indicate 77% received medication for diabetes (73% for Medicare, 75% for Medicaid, and 82% for commercially insured). Rates ranged from 70% treated with medication in New Jersey to 82% in Utah. Among Medicare patients, 76% of the age 65–74 population had claims for diabetes medication versus 71% of the population age 75 and older. Overall, 46% had claims for metformin, 23% had claims for insulin, and 29% had claims for sulfonylureas, with smaller percentages of patients having claims for other diabetes single therapy or combination medications. Rates varied by state even within each insurance type. While 90% of commercially insured people with diagnosed T2DM in California received medication, the percentages were substantially lower in Northeastern states including Maine (74%), New Jersey (76%), Massachusetts (77%), and Rhode Island and New Hampshire (78%). There was even greater state variation in percentage receiving medication within the Medicaid population-ranging from 84% in Connecticut to 44% in Arizona.

Diagnosis codes and medication claims suggest 80% of diagnosed T2DM adults had hypertension (controlled or uncontrolled, ranging from 91% for Medicare to 61% for Medicaid), and 76% had hyperlipidemia (ranging from 83% for Medicare to 43% for Medicaid). Medication claims for these diagnosed T2DM patients indicate 63% received ACE inhibitors/ARBs, 47% received other anti-hypertensive medications (e.g., diuretics), and 58% received statins.

### Poorly controlled diabetes, sequelae, and costs

Among diagnosed patients receiving diabetes medications, 43% had at least one medical claim during the year where an ICD-9 code indicated the patient’s diabetes was poorly controlled (Table 3). This estimate was similar across all three insurance types. The presence of diabetes sequelae was higher among the population with uncontrolled diabetes relative to the population with no indication of uncontrolled diabetes. Prevalence of neurological conditions was 14 percentage points higher among the uncontrolled diabetes population compared to the population with no indication of uncontrolled diabetes. Similarly, among the uncontrolled population there was higher prevalence of renal failure (14 percentage points higher), “other” complications (13 percentage points higher), peripheral vascular complications (11 percentage points higher), and endocrine/metabolic complications (10 percentage points higher). The Medicare population with diagnosed T2DM had higher rates of diabetes sequelae compared to the commercially insured and Medicaid populations. For example, 36% of the Medicare population with no indication of uncontrolled diabetes still had claims indicating presence of neurological complications-versus 17% prevalence among the Medicaid and 12% among the commercially insured populations. Among the population with indications of uncontrolled diabetes, prevalence of neurological complications was 52, 31, and 23% for the Medicare, Medicaid, and commercially insured populations, respectively. Differences in complication prevalence across insurance type and by controlled/uncontrolled status likely contributed to the large differences in average annual medical costs.

**Table 3:** Complications and expenditures for adults with type 2 diabetes receiving diabetes medications

National metrics	Total insured	Commercial	Medicare	Medicaid
Controlled diabetes	7,440,000	3,480,000	3,270,000	690,000
Controlled (%)	57%	59%	56%	58%
Presence of diabetes sequelae				
Neurological complications	24%	12%	36%	17%
Peripheral vascular disease	19%	8%	32%	12%
Cardiovascular disease	80%	71%	93%	61%
Renal complications	28%	17%	40%	20%
Endocrine/metabolic complications	72%	70%	81%	38%
Ophthalmic complications	35%	21%	51%	22%
Other complications	17%	14%	20%	19%
Foot problems	7%	3%	10%	7%
Total expenditures	$17,500	$13,160	$21,280	$19,000
Medical expenditures	$12,980	$9,630	$15,900	$14,110
Rx expenditures	$4,520	$3,530	$5,380	$4,890
Uncontrolled diabetes	5,550,000	2,450,000	2,590,000	510,000
Uncontrolled (%)	43%	41%	44%	42%
Presence of diabetes sequelae				
Neurological complications	38%	23%	52%	31%
Peripheral vascular disease	30%	13%	46%	20%
Cardiovascular disease	88%	80%	96%	76%
Renal complications	42%	27%	56%	33%
Endocrine/metabolic complications	82%	82%	88%	51%
Ophthalmic complications	43%	28%	58%	33%
Other complications	30%	24%	34%	35%
Foot problems	12%	7%	17%	12%
Total expenditures	$22,410	$19,730	$24,440	$23,490
Medical expenditures	$17,140	$14,880	$18,900	$17,830
Rx expenditures	$5,270	$4,850	$5,540	$5,660
Uncontrolled impact (percentage point or $ change)				
Presence of diabetes sequelae				
Neurological complications	14%	11%	16%	14%
Peripheral vascular disease	11%	5%	14%	8%
Cardiovascular disease	8%	9%	4%	15%
Renal complications	14%	10%	16%	13%
Endocrine/metabolic complications	10%	12%	7%	13%
Ophthalmic complications	8%	7%	7%	11%
Other complications	13%	10%	14%	16%
Foot problems	5%	3%	7%	6%
Total expenditures	$4,910	$6,570	$3,160	$4,490
Medical expenditures	$4,160	$5,250	$3,000	$3,720
Rx expenditures	$750	$1,320	$160	$770
Controlled (age-sex-adjusted to uncontrolled population)				
Total expenditures	$17,550	$13,050	$21,010	$19,130
Medical expenditures	$12,990	$9,560	$15,630	$14,170
Rx expenditures	$4,560	$3,490	$5,380	$4,960
Uncontrolled impact (age-sex-adjusted)				
Total expenditures	$4,860	$6,680	$3,430	$4,360
Medical expenditures	$4,150	$5,320	$3,270	$3,660
Rx expenditures	$710	$1,360	$160	$700

Total annual health care expenditures for the treated population with controlled diabetes averaged $17,500 per patient (ranging from $13,160 for commercially insured patients to $21,280 for Medicare patients). This includes both diabetes- and non-diabetes-related expenditures. In comparison, annual health care expenditures averaged $22,410 for the uncontrolled population (ranging from $19,730 for commercially insured patients to $24,440 for Medicare patients). Adjusting the age-sex distribution of the controlled population to match demographics of the uncontrolled population suggests that patients with uncontrolled diabetes have higher annual health care expenditures averaging $4,860 per T2DM patient-including $4,150 in higher medical expenditures and $710 in higher prescription expenditures (with higher prescription expenditures associated primarily with higher costs for insulin). Average expenditure differences between controlled and uncontrolled were highest for the commercially insured population ($6,680), followed by the Medicaid population ($4,360) and Medicare population ($3,430). Average difference in medical costs between controlled and uncontrolled patients was highest for Nevada ($7,800) and Nebraska ($7,530), while New Mexico ($2,080) and California ($2,410) had the smallest excess costs (Additional file 2).

Key findings are summarized in Table 4. Close to 25.3 million adults with diabetes have medical insurance coverage, but among these only 18.3 million are diagnosed. Of the estimated 6.9 million undiagnosed, at least 6.3 million are T2DM (suggesting 27% of people with T2DM are undiagnosed). The large majority (92%) of diagnosed cases are T2DM, and among the T2DM population 77% have medication claims indicating use of diabetes medicine. Even if all type 1 patients used diabetes medication (insulin), these numbers suggest that at most 79% of the insured population with diabetes used diabetes medication-a number below the 86% estimate from self-report national survey data for all adults with diagnosed diabetes, both insured and uninsured [2].

**Table 4:** Summary metrics for diabetes diagnosis, treatment, and control

Key metrics	Total insured	Commercial	Medicare	Medicaid
Total with diabetes	25,260,000	11,400,000	11,430,000	2,430,000
Total undiagnosed	6,920,000	3,110,000	3,150,000	660,000
Percent of type 2 diabetes patients undiagnosed^a^	27%	27%	28%	27%
Diagnosed	18,340,000	8,290,000	8,280,000	1,770,000
Diagnosed type 1	1,450,000	1,070,000	200,000	180,000
Diagnosed type 2	16,890,000	7,220,000	8,080,000	1,590,000
% not receiving diabetes medication	23%	18%	27%	25%
Not receiving diabetes medication	3,900,000	1,290,000	2,220,000	390,000
Receiving diabetes medication	12,990,000	5,930,000	5,860,000	1,200,000
Poorly controlled diabetes	5,550,000	2,450,000	2,590,000	510,000
Controlled diabetes	7,440,000	3,480,000	3,270,000	690,000
% poorly controlled	43%	41%	44%	42%
Average increase in medical expenditures for uncontrolled diabetes	$4,860	$6,680	$3,430	$4,260

^a^Undiagnosed cases in each age group are assumed to have the same proportion type 2 as diagnosed cases in each age group

## Discussion

Approximately 84% of adults in 2012 with diagnosed or undiagnosed diabetes had medical insurance. Presumably these insured adults had access to care, but still an estimated 27% of diabetes cases remained undiagnosed. The new guidelines from the US Preventive Services Task Force for diabetes screening of asymptomatic adults recommend screening for adults with risk factors for diabetes (including all adults aged 40 to 70 years who are overweight or obese, or who have one or more other known risk factors for diabetes such as family history of diabetes) [9]. If adopted and used by practitioners, increased targeted screening could reduce the rate of undiagnosed cases among the insured population. The Congressional Budget Office estimates that the Affordable Care Act will by 2017 result in 26 million people receiving medical coverage who would otherwise be uninsured [12]. Together, increased insurance coverage and adoption and use of broader screening guidelines could reduce the number of undiagnosed diabetes cases.

Still, among adults with diagnosed T2DM, some appear to have inadequate disease management. Approximately 80% of these adults have diagnosed hypertension (whether treated or untreated), while approximately 63% of T2DM adults have medication claims for ACE inhibitors/ARBs and 47% have medication claims for diuretics or other anti-hypertensive medications. Approximately 76% of these adults have hyperlipidemia, yet only 58% have medication claims for statins (though other medications might be used to treat hyperlipidemia). Only 82% of these patients had at least one A1c test during the year (guidelines are to receive two or more) [1]. Half received a urinary albumin test (guidelines call for annual screening) [1].

The need for diabetes medications will differ by patient and can change over time. Approximately 77% of diagnosed T2DM patients had claims for diabetes medications. Among those receiving medication, 43% had medical claims indicating uncontrolled diabetes. Patients with indications of poor glycemic control had higher prevalence of complications and $4,860 higher annual health care expenditures compared to a similar population with controlled glycemic levels.

This study reports a large set of health outcomes for patients with T2DM at the national and state levels. We found few studies with similar scope to compare with at the state level. However, at the national level, our estimates are comparable with other estimates. For instance, the American Diabetes Association [13] reports that 90 to 95% of diagnosed diabetes cases are T2DM, while our estimate was 92%, ranging from 87% among privately insured to 98% among Medicare beneficiaries with diabetes. CDC reported that approximately 81% of diagnosed diabetes (including type 1 and T2DM) patients aged 18 and above in 2011 were treated with either oral drugs, insulin, or both based on self-reported data, while we found that 77% (T2DM only) receive medication [14]. A 2013 study using electronic medical records found that in the period between 2011 and 2012, 81.6% of diabetes patients had an A1c test (almost identical to our finding of 82% receiving annual A1c test among T2DM patients). This same study also found that 39% of the diabetes patients did not have their diabetes under control defined as A1c < 7, while we found 43% uncontrolled based on diagnosis codes [15].

### Study strengths and limitations

This study analyzed medical claims for large samples of commercially insured, Medicare, and Medicaid populations to provide insight on treatment patterns, prevalence of diabetes-related complications, and medical and prescription medicine expenditures. The use of medical claims provides a comparison to diabetes management statistics based on self-reported surveys. Study limitations include the following:

Analysis of medical claims was restricted to a fee-for-service population as medical claims are often incomplete for patients in capitated managed care plans. It is unclear how this might bias study results. Medicare patients with poorer health might choose a fee-for-service plan over a managed care plan because it gives them greater access to physicians not in a managed care network, but this is unlikely to affect diabetes detection, treatment, and control. Medicaid patients often have less ability to select whether they are in a managed care or fee-for-service type arrangement (with plan type often determined by state policy).

Uncontrolled status was determined by diagnosis code and not independent lab results. While laboratory results are available for a subset of the commercially insured T2DM patients, Medicare and Medicaid files do not contain laboratory results. To create a consistent definition of diabetes control across insurance types, we identified patients with controlled diabetes as those who were not diagnosed with uncontrolled diabetes (ICD-9 code 250.×2 or 250.×3) during the study year. For the subset of commercially insured T2DM patients for which we had both ICD-9 and A1c information, we performed additional correlation analyses to assert the strength of the association between the two measures of diabetes control. We found a statistically significant and positive correlation between ICD-9- and A1c-based case identification measures (Spearman rank correlation coefficients of 0.22 [*P* < 0.001] for A1c > 9% as uncontrolled). However, the low correlation suggests that ICD-9 indications of uncontrolled diabetes, as currently coded, are a weak proxy for actual A1c results. Chi-square statistics were also significant. Patients who are sicker tend to have more touch points with the health care system and thus generate more medical claims, so there is a higher likelihood that they might be categorized as having uncontrolled diabetes. This could bias high the estimated expenditure impact associated with uncontrolled diabetes.

Medical claims data were unavailable for care provided through the Veterans Health Administration, Indian Health Services, and other providers. Estimates for the number of adults age 65 or older with diabetes are counted under Medicare-including veterans receiving care through the VHA. Estimates for the number of adults under age 65 with diabetes who receive health care through a government-sponsored program are counted under Medicaid-including Medicaid dual-eligible patients, veterans receiving care through the VHA, and other government-sponsored plans such as IHS.

The OptumInsight dNHI claims data, while nationally representative of the privately insured patient population, might not be representative for some states where United Healthcare and other insurers represented in dNHI have smaller market share. Consequently, for this analysis we estimated outcomes using dNHI data in each state by age and gender and then weighted outcomes based on the age and gender distribution of privately insured people in each state in our constructed state population file. States with small sample size in the dNHI are Alaska, Hawaii, Montana, South Dakota, Vermont, and Wyoming. When the sample was less than *n* = 30 patients for a particular age-gender strata, we used national outcomes rather than state outcomes for that demographic group. Hence, for these states the metrics reported in this paper for the commercially insured population are biased toward the national average.

The analysis relied in part on self-reported data collected through surveys (American Community Survey, Behavioral Risk Factor Surveillance System, and National Health and Nutrition Examination Survey). Self-reported household income, body weight, smoking status, and other patient characteristics can be unreliable.

For some data sources we combined data from multiple years between 2008 and 2012 to increase sample size. Combining data across years makes the implicit assumption that diagnosis rates and the proportion of diagnosed diabetes cases that are T2DM remained relatively constant over this period.

These data limitations suggest possible areas for improvement. For example, a centralized source for patient information-such as all-payer claims databases that some states are developing-will facilitate comparing diabetes-related outcomes across geographic locations and payers, thus facilitating the identification of populations doing well (to identify possible best practices) and identifying populations doing poorly (to identify needs). This study used ICD-9 diagnosis codes and self-reported data in surveys, but such sources can be unreliable. Increased availability of data from electronic health records could provide more reliable data for future benchmarking.

## Conclusions

The large number of patients with undiagnosed diabetes and large numbers of diabetes patients not receiving medication (for diabetes, hypertension, and hyperlipidemia) or monitoring exams indicate a need for greater commitment by providers to closely monitor their patients and be more aggressive in following standards of medical care. These findings underscore that, among an insured population that presumably has good access to professional care, too many people have undiagnosed diabetes and those whose diabetes is diagnosed often require better management of their disease. The uninsured are more likely than their insured peers with similar demographics to be undiagnosed, and having insurance is associated with improved control of diabetes [16–18]. Consequently, expanding insurance coverage through the Affordable Care Act is part of the solution to the lack of diagnosis and diabetes control challenge. While Medicare patients are the most costly overall to treat, our findings suggest the medical expenditure gap between controlled and uncontrolled diabetes is higher for commercially insured patients than for government-insured patients. Additional research could help identify why diabetes-related outcomes differ substantially by state and insurance type, with the goal to inform policies or practices that could help improve diabetes outcomes among low-performing populations.

## Additional files

Additional file 1: Technical documentation. (DOCX 37 kb)
Additional file 2: State estimates. (DOCX 94 kb)

## Abbreviations

ACE: Angiotensin-converting enzyme
ACS: American Community Survey
ARB: Angiotensin II receptor blockers
BRFSS: Behavioral Risk Factor Surveillance System
CDC: Centers for Disease Control and Prevention
dNHI: De-identified Normative Health Information
HEDIS: The Healthcare Effectiveness Data and Information Set
ICD-9: International Classification of Diseases, Ninth Revision
NNHS: National Nursing Home Survey
T2DM: Type 2 diabetes
