# Supplementary material for: Type 2 diabetes detection and management among insured adults
Source: Popul Health Metr. 2016 Nov 21;14:43. doi: 10.1186/s12963-016-0110-4 (PMC5117523; doi:10.1186/s12963-016-0110-4)
Supplement: Additional file 1: — Technical documentation. (DOCX 37 kb) [file 12963_2016_110_MOESM1_ESM.docx]

# Additional file 1: Technical documentation

## National Health and Nutrition Examination Survey Analysis for Undiagnosed Diabetes

In the NHANES sample with lab results, most adults received a single test for diabetes – hemoglobin A1c (A1c), fasting plasma glucose test (FPG), or 2-hour oral glucose tolerance test (OGTT) – although some adults received multiple tests. Consistent with the approach used by the Centers for Disease Control and Prevention to estimate national rates of diabetes (diagnosed and undiagnosed), we indicate presence of diabetes if either A1c or FPG exceeds diabetes thresholds: A1c ≥ 6.5 percent, or FPG ≥ 126 mg/dl.^[[1]](#footnote-1)^

Among the 503 adults who were categorized as having previously undiagnosed diabetes (Table A-1), the largest number were identified based on A1c alone (n=223), OGTT alone (n=146), or FPG alone (n=95). The remaining 185 adults were diagnosed based on a combination of tests. Unlike a clinical setting, NHANES does not conduct follow-up tests to confirm the original test results.

Table A-1: Diagnosis tests used to identify adults with previously undiagnosed diabetes

| **Diagnosis test** | **Number identified** | **Percent of total (%)** |
| --- | --- | --- |
| A1c alone | 244 | 49 |
| FPG alone | 141 | 28 |
| A1c & FPG | 118 | 23 |
| Total | 503 | 100 |

Notes:

Statistically significant predictors of having undiagnosed diabetes (among adults who indicate never having been told by a health professional that they have diabetes) are age, non-white, Hispanic, hypertension, cardiovascular disease, no history of arthritis, excess body weight, and uninsured (Table A-2). Some patient characteristics included as explanatory variables (such as household income) are self-reported and thus potentially unreliable. Controlling for patient risk factors, probability of having undiagnosed diabetes did not differ between the 2009–2010 and 2011–2012 waves of NHANES.

Table A-2: Logistic regression results predicting previously undiagnosed diabetes

| **Explanatory variables** | **Odds ratio** | **95% Confidence limits** | |
| --- | --- | --- | --- |
| Age | 1.372 | 1.298 | 1.451 |
| Male | 1.145 | 0.964 | 1.360 |
| Race/ethnicity (vs non-Hispanic White) |  |  |  |
| Non-Hispanic Black | 1.763 | 1.355 | 2.293 |
| Non-Hispanic Other | 3.034 | 2.159 | 4.265 |
| Hispanic | 2.254 | 1.759 | 2.889 |
| Presence of disease |  |  |  |
| Asthma | 0.886 | 0.665 | 1.181 |
| Arthritis | 0.905 | 0.723 | 1.133 |
| History of myocardial infarction | 0.966 | 0.620 | 1.505 |
| History of stroke | 0.758 | 0.468 | 1.227 |
| History of cancer | 1.027 | 0.751 | 1.404 |
| Hypertension | 1.552 | 1.251 | 1.925 |
| High cholesterol | 1.144 | 0.932 | 1.405 |
| Cardiovascular disease | 1.527 | 1.073 | 2.172 |
| Current smoker | 1.149 | 0.895 | 1.474 |
| Overweight (vs normal weight) | 1.816 | 1.342 | 2.457 |
| Obese (vs normal weight) | 4.599 | 3.457 | 6.118 |
| Has medical insurance | 0.683 | 0.533 | 0.877 |
| Covered by Medicaid | 1.056 | 0.745 | 1.499 |
| Household income level | 0.998 | 0.995 | 1.001 |
| Survey year 2011–2012 vs 2009–2010 | 1.018 | 0.840 | 1.233 |

Note: Sample size was 9,943. Overall Likelihood Ratio probability of Chi-Squared < 0.0001. Percent Concordant = 78.1.

## Diabetes sample inclusion criteria and identification algorithm for medical claims analysis

The following describes the inclusion and exclusion criteria with the medical claims analysis to distinguish type 2 from type 1 diabetes patients, procedure codes used to identify HEDIS-related clinical metrics, and diagnosis codes used to identify diabetes complication categories.

**For inclusion in the medical claims analysis, patient must meet all the following criteria:**

- Evidence of diabetes (identified with ICD-9-CM diagnosis codes 250.xx) in the 6 months pre-index period.
- Continuously enrolled in commercial coverage with no more than one gap in enrollment of up to 45 days during the measurement year. In Medicare and Medicaid, only beneficiaries with 12 months’ continuous enrollment were considered.
- Patients age 18 years or older.
- No evidence of gestational diabetes during the pre-index, baseline, and follow-up periods.

**Type 2 diabetes identification algorithm**

Type 2 diabetes is defined as a patient who meets the following criteria using data from the 180 days prior to and following the index date:

- - ≥1 medical claim for type 2 diabetes (ICD-9-CM diagnosis code of 250.x0 or 250.x2) and no claims for type 1 diabetes mellitus, identified with ICD-9-CM diagnosis codes 250.x1 or 250.x3. Diagnosis codes in the primary or secondary positions used;

OR

- - If medical claims for both type 1 and type 2 diabetes, the patient must meet one of the following:
    1. ≥1 claim for an oral anti-diabetic (OAD) medication including sulfonylureas, metformin, thiazolidinediones, α-glucosidase inhibitors, meglitinide derivatives, DPP-4 inhibitors, or combination of OADs with insulin or non-insulin injectable;

OR

- - 1. If no claims for OADs, the patient must have 4 or more claims for 250.xx with a valid 5th digit AND the number of claims for Type 2 diabetes (250.x0, 250.x2) must exceed the number of claims for Type 1 diabetes (250.x1, 250.x3);

OR

- - If no medical claims for 250.xx with a valid 5th digit, then the patient must have ≥1 claim for an OAD AND a claim for an injectable antidiabetic medication (GLP-1RA, pramlintide or insulin), and no evidence of medical claims identifying T1DM patient in the previous year;

OR

- - If no medical claims for 250.xx with a valid 5th digit and no claims for injectable antidiabetic medications, then the patient must have ≥1 claim for an OAD AND no medical claims with any of the following ICD-9 codes: 256.4, 272.6, 277.7, 648.8x, 790.2x. Codes in any position were used.

## Procedure and diagnosis codes

Table A-3 lists procedure codes used to identify whether a particular exam was provided.

Table A-3: Procedure codes used to identify HEDIS-related clinical metrics

| **Type of exam/use** | **CPT** | **CPT category II** | **LOINC** | **HCPCS** | **ICD-9 procedure** |
| --- | --- | --- | --- | --- | --- |
| HbA1c | 83036, 83037 | 3044F, 3045F, 3046F, 3047F |  |  |  |
| LDL cholesterol | 80061, 83700, 83701, 83704, 83721 | 3048F, 3049F, 3050F | 2089-1, 12773-8, 13457-7, 18261-8, 18262-6, 22748-8, 39469-2, 49132-4, 55440-2 |  |  |
| Eye exam | 67028, 67030, 67031, 67036, 67039-67043, 67101, 67105, 67107, 67108, 67110, 67112, 67113, 67121, 67141, 67145, 67208, 67210, 67218, 67220, 67221, 67227, 67228, 92002, 92004, 92012, 92014, 92018, 92019, 92134, 92225-92228, 92230, 92235, 92240, 92250, 92260, 99203-99205, 99213-99215, 99242-99245 | 2022F, 2024F, 2026F, 3072F |  | S0620, S0621, S0625, S3000 | 141, 142, 143, 144, 145, 149, 9502, 9503, 9504, 9511, 9512, 9516 |
| Blood pressure |  | 3074F, 3075F, 3077F, 3078F, 3079F, 3080F |  |  |  |
| Macroalbumin | 81000, 81001, 81002, 81003, 81005 | 3062F |  |  |  |
| ACEI/ARB |  | 4009F |  |  |  |
| Nephropathy | 82042, 82043, 82044, 84156 | 3060F, 3061F | 1753-3, 1754-1, 1755-8, 1757-4, 2887-8, 2888-6, 2889-4, 2890-2, 9318-7, 11218-5, 12842-1, 13705-9, 13801-6, 14585-4, 14956-7, 14957-5, 14958-3, 14959-1, 18373-1, 20621-9, 21059-1, 21482-5, 26801-1, 27298-9, 30000-4, 30001-2, 30003-8, 32209-9, 32294-1, 32551-4, 34366-5, 35663-4, 40486-3, 40662-9, 40663-7, 43605-5, 43606-3, 43607-1, 44292-1, 47558-2, 49023-5, 50949-7, 53121-0, 53530-2, 53531-0, 53532-8, 56553-1, 57369-1, 58448-2, 58992-9, 59159-4, 60678-0, 63474-1 |  |  |

Table A-4 lists symptoms included in the diabetes-related complication groups – though not all complications necessarily resulted from diabetes (as diabetes is only one of multiple risk factors for many of these conditions). We selected the list of key diabetes complication measures based on a review of literature, especially the HEDIS Comprehensive Diabetes Care measures and the Economic Cost of Diabetes in the US in 2012 study by American Diabetes Association.^[[2]](#footnote-2)^ New research suggests adding other conditions to the list of diabetes complications. For example, recent research reports that 75% of men with type 2 diabetes experience erectile dysfunction and twice the odds of having undiagnosed diabetes.^[[3]](#footnote-3)^

Table A-4: Diabetes complication groups

| **Complication group** | **ICD-9 codes** |
| --- | --- |
| ***Neurological symptoms*** |  |
| Diabetes with neurological complications | 250.6 |
| Peripheral autonomic neuropathy | 337.1 |
| Mononeuritis of upper limb and mononeuritis multiplex | 354 |
| Mononeuritis of lower limb | 355 |
| Other specified idiopathic peripheral neuropathy | 356.8 |
| Polyneuropathy in diabetes | 357.2 |
| Myasthenic syndromes in diseases classified elsewhere | 358.1 |
| Subarachnoid hemorrhage | 430 |
| Intracerebral hemorrhage | 431 |
| Other and unspecified intracranial hemorrhage | 432 |
| Occlusion of stenosis of pre-cerebral arteries | 433 |
| Occlusion of cerebral arteries | 434 |
| Transient ischemic attack | 435 |
| Ill-defined cerebrovascular disease | 436–437 |
| Late effects of cerebrovascular disease | 438 |
| Arthropathy associated w/neurological disorders | 713.5 |
| Neuralgia, neuritis, and radiculitis, unspecified | 729.2 |
| ***Peripheral vascular disease*** |  |
| Diabetes with peripheral circulatory disorders | 250.7 |
| Atherosclerosis | 440 |
| Raynaud’s syndrome | 443 |
| Thromboangiitis obliterans | 443.1 |
| Other arterial dissection | 443.2 |
| Erythromelalgia | 443.82 |
| Embolism and thrombosis, structure of artery | 444 |
| Phlebitis and thrombophlebitis | 451 |
| Portal vein thrombosis | 452 |
| Other venous embolism and thromboembolism | 453 |
| Varicose veins of lower extremities | 454 |
| Other disorders of circulatory system | 459 |
| Ulcer of lower limb, unspecified | 707.1 |
| Ulcer of thigh, calf, and ankle | 707.11–707.13 |
| Ulcer of other part of lower limb | 707.19 |
| Pressure ulcer stages | 707.2 |
| Chronic ulcer of other specified sites | 707.8 |
| Chronic ulcer of unspecified site | 707.9 |
| Traumatic amputation of thumb, fingers, arm, and hand | 885–887 |
| Pressure ulcer | 707.0 |
| ***Cardiovascular disease*** |  |
| Essential hypertension | 401 |
| Hypertensive heart disease | 402 |
| Hypertensive chronic kidney disease | 403 |
| Hypertensive heart and chronic kidney disease | 404 |
| Secondary hypertension | 405 |
| Acute myocardial infarction | 410 |
| Other acute and subacute forms of ischemic heart disease | 411 |
| Old myocardial infarction | 412 |
| Angina | 413 |
| Other chronic ischemic heart disease | 414 |
| Cardiomyopathy | 425 |
| Conduction disorders | 426 |
| Cardiac dysrhythmias | 427 |
| Heart failure | 428 |
| Myocardial degeneration | 429.1 |
| Cardiovascular disease, unspecified | 429.2 |
| Cardiomegaly | 429.3 |
| Aortic aneurysm and dissection | 441 |
| Other aneurysm | 442 |
| Hypotension | 458 |
| ***Renal complications*** |  |
| Diabetes and renal complications | 250.4 |
| Acute glomerulonephritis | 580 |
| Nephrotic syndrome | 581 |
| Chronic glomerulonephritis | 582 |
| Nephritis and nephropathy, not specified as acute or chronic | 583 |
| Acute renal failure | 584 |
| Chronic renal failure (ESRD) | 585 |
| Renal failure, unspecified | 586 |
| Renal sclerosis, unspecified | 587 |
| Disorders resulting from impaired renal function | 588 |
| Infections of kidney | 590 |
| Other disorders of kidney and ureter | 593 |
| Cystitis | 595 |
| Other disorders of bladder | 596 |
| Urinary tract infection | 599.0 |
| Proteinuria | 791.0 |
| ***Endocrine/metabolic complications*** |  |
| Dwarfism-obesity syndrome | 259.4 |
| Other specified endocrine disorders | 259.8 |
| Lancereaux’s disease | 261 |
| Galactosemia | 271.1 |
| Renal glycosuria | 271.4 |
| Hypertriglyceridemia | 272.1 |
| Mixed hyperlipidemia | 272.2 |
| Hyperchylomicronemia | 272.3 |
| Other and unspecified hyperlipidemia | 272.4 |
| Lipidoses | 272.7 |
| Macroglobulinemia | 273.3 |
| Hyperkalemia | 276.7 |
| Glycogenosis | 271.0 |
| Hypercholesterolemia | 272.0 |
| Disorders of iron metabolism | 275.0 |
| ***Ophthalmic complications*** |  |
| Diabetes with ophthalmic complications | 250.5 |
| Other retinal disorders | 362 |
| Vascular disorders of the iris and ciliary body | 364.4 |
| Glaucoma | 365 |
| Cataract | 366 |
| Visual disturbances | 368 |
| Blindness and low vision | 369 |
| Disorders of the optic nerve and visual pathways | 377 |
| Acute and subacute iridocyclitis | 364.0 |
| ***Other complications of diabetes*** |  |
| Candidiasis of vulva and vagina | 112.1 |
| Candidiasis of skin and nails | 112.3 |
| Diabetes with other specified manifestations | 250.8 |
| Diabetes with unspecified complication | 250.9 |
| Infective otitis externa | 380.1 |
| Other and unspecified noninfectious gastroenteritis and colitis | 558.9 |
| Impotence of organic origin | 607.84 |
| Cellulitis and abscess of finger | 681.0, 681.9, |
| Cellulitis and abscess of unspecified digit | 681.9 |
| Other cellulitis and abscess | 682.1– 682.5, 682.8, 682.9 |
| Degenerative skin disorders | 709.3 |
| Other bone involvement in disease classified elsewhere | 731.8 |
| Bacteremia | 790.7 |
| Coxsackie virus | 079.2 |
| Cellulitis and abscess of face | 682.0 |
| ***Complications related to the foot*** |  |
| Atherosclerosis of native arteries of the extremities, unspecified | 440.2 |
| Atherosclerosis with ulceration | 440.23 |
| Atherosclerosis with gangrene | 440.24 |
| Generalized and unspecified atherosclerosis | 440.9 |
| Peripheral angiopathy in diseases classified elsewhere | 443.81 |
| Other specified peripheral vascular diseases | 443.89 |
| Peripheral vascular disease, unspecified | 443.9 |
| Stricture of artery | 447.1 |
| Unspecified circulatory system disorder | 459.9 |
| Cellulitis and abscess of toe, foot, and leg | 681.1, 682.6, 682.7 |
| Ulcer of heel, mid-foot, and other parts of the foot | 707.14, 707.15 |
| Chronic osteomyelitis of the foot | 730.17 |
| Gangrene and amputations | 785.4 |
| Traumatic amputation of toe(s), foot, and leg | 895–897 |

We identified medication use for diabetes using NDC therapeutic classes. Antidiabetic agents included oral antidiabetic drugs (OAD), including alpha-glucosidase inhibitors, meglitinides, biguanides, sulfonylureas, thiazolidinediones, and dipeptidyl peptidase-4 inhibitors, and antidiabetic combinations, insulin mixes, long-acting insulins, and non-insulin injectable drugs (GLP-1 receptor agonists). Rapid-acting insulin was excluded due to the uncertainty of the real days of supply. Days of supply for insulin and non-insulin injectable drugs were adjusted due to titration using existing methods.^[[4]](#footnote-4)^ Drug lists were constructed using Healthcare Effectiveness Data and Information Set (HEDIS) approved diabetes drugs by 2011 (the National Drug Code list available upon request).

1. Centers for Disease Control and Prevention. National Diabetes Statistics Report, 2014: Data Sources, Methods, and References for Estimates of Diabetes and Its Burden in the United States. <http://www.cdc.gov/diabetes/pdfs/data/2014-report-national-diabetes-statistics-report-data-sources.pdf> [↑](#footnote-ref-1)
2. American Diabetes Association. Economic costs of diabetes in the US in 2012. Diabetes care 2013. [↑](#footnote-ref-2)
3. Skeldon SC, Detsky AS, Goldenberg SL, Law MR. Erectile Dysfunction and Undiagnosed Diabetes, Hypertension, and Hypercholesterolemia. Ann Fam Med. 2015 Jul-Aug;13(4):331-5. [↑](#footnote-ref-3)
4. Buysman, E., et al., Adherence and persistence to a regimen of basal insulin in a pre-filled pen compared to vial/syringe in insulin-naive patients with type 2 diabetes. *Curr Med Res Opin*, 2011. 27(9): p. 1709-17.

   Malmenas, M., J.R. Bouchard, and J. Langer, Retrospective real-world adherence in patients with type 2 diabetes initiating once-daily liraglutide 1.8 mg or twice-daily exenatide 10 mug. *Clin Ther*, 2013. 35(6): p. 795-807. [↑](#footnote-ref-4)
