# Supplementary material for: Type 2 diabetes detection and management among insured adults
Source: Popul Health Metr. 2016 Nov 21;14:43. doi: 10.1186/s12963-016-0110-4 (PMC5117523; doi:10.1186/s12963-016-0110-4)
Supplement: Additional file 2: — State estimates. (DOCX 94 kb) [file 12963_2016_110_MOESM2_ESM.docx]

# Additional file 2: State estimates

Table B-1. Diabetes population metrics by state: Adults age 20+, 2012

| **State** | **Total Diabetes ^a,b^** | **# Undiagnosed Diabetes ^b^** | **Diagnosed** | | | | | | |
| --- | --- | --- | --- | --- | --- | --- | --- | --- | --- |
|  |  |  | **# Uninsured** | **Insured** | | | | | |
|  |  |  |  | **# Insured** | **Type 2** | | | | |
|  |  |  |  |  | **# Untreated with Rx** | **Treated with Rx** | | | |
|  |  |  |  |  |  | **# Treated** | **# Uncontrolled** | **# Controlled** | **Uncontrolled cost impact ($)** |
| AK | 48,600 | 18,400 | 4,500 | 25,700 | 5,100 | 18,600 | 7,100 | 11,500 | 4,210 |
| AL | 547,900 | 126,900 | 80,000 | 341,000 | 63,700 | 250,400 | 103,400 | 147,000 | 3,620 |
| AR | 314,100 | 75,400 | 53,100 | 185,600 | 35,400 | 135,100 | 51,700 | 83,400 | 4,630 |
| AZ | 637,200 | 171,500 | 64,600 | 401,100 | 100,200 | 266,100 | 128,400 | 137,700 | 6,450 |
| CA | 3,433,800 | 1,021,400 | 439,000 | 1,973,400 | 362,200 | 1,453,600 | 547,700 | 905,900 | 2,490 |
| CO | 355,000 | 117,600 | 34,300 | 203,100 | 36,100 | 148,100 | 64,000 | 84,100 | 5,890 |
| CT | 307,000 | 92,500 | 24,600 | 189,900 | 43,600 | 129,200 | 53,800 | 75,400 | 6,300 |
| DC | 48,300 | 16,700 | 2,500 | 29,100 | 5,700 | 19,600 | 9,500 | 10,100 | 7,160 |
| DE | 98,700 | 25,000 | 7,000 | 66,700 | 15,000 | 45,500 | 20,000 | 25,500 | 6,000 |
| FL | 2,303,300 | 578,600 | 310,700 | 1,414,000 | 370,700 | 941,500 | 445,400 | 496,100 | 4,740 |
| GA | 951,500 | 241,100 | 144,400 | 566,000 | 114,900 | 402,100 | 180,000 | 222,100 | 5,200 |
| HI | 130,600 | 46,300 | 5,200 | 79,100 | 19,300 | 54,100 | 21,800 | 32,300 | 2,490 |
| IA | 258,500 | 75,200 | 20,300 | 163,000 | 30,500 | 121,800 | 35,900 | 85,900 | 5,120 |
| ID | 134,100 | 35,700 | 18,500 | 79,900 | 15,300 | 57,300 | 20,300 | 37,000 | 4,710 |
| IL | 1,168,500 | 340,500 | 162,900 | 665,100 | 129,000 | 478,900 | 204,400 | 274,500 | 5,710 |
| IN | 638,300 | 159,900 | 81,300 | 397,100 | 74,300 | 291,300 | 116,800 | 174,500 | 5,610 |
| KS | 265,100 | 69,100 | 29,700 | 166,300 | 31,200 | 122,500 | 46,500 | 76,000 | 5,230 |
| KY | 486,100 | 107,600 | 71,800 | 306,700 | 61,400 | 225,300 | 83,800 | 141,500 | 5,520 |
| LA | 500,600 | 124,400 | 96,600 | 279,600 | 52,200 | 208,700 | 87,300 | 121,400 | 4,650 |
| MA | 606,700 | 162,400 | 34,800 | 409,500 | 93,900 | 276,900 | 117,100 | 159,800 | 6,880 |
| MD | 564,600 | 155,800 | 43,300 | 365,500 | 82,800 | 252,100 | 124,300 | 127,800 | 5,640 |
| ME | 135,300 | 35,600 | 13,800 | 85,900 | 21,300 | 58,400 | 22,000 | 36,400 | 3,610 |
| MI | 1,002,400 | 258,700 | 121,000 | 622,700 | 150,500 | 424,100 | 176,300 | 247,800 | 5,010 |
| MN | 400,600 | 126,300 | 24,900 | 249,400 | 53,000 | 172,100 | 50,500 | 121,600 | 4,250 |
| MO | 567,100 | 152,000 | 68,300 | 346,800 | 68,300 | 247,900 | 113,300 | 134,600 | 4,580 |
| MS | 339,900 | 79,000 | 58,700 | 202,200 | 36,100 | 153,100 | 64,600 | 88,500 | 2,980 |
| MT | 75,400 | 26,200 | 10,000 | 39,200 | 7,900 | 28,400 | 11,000 | 17,400 | 4,940 |
| NC | 987,700 | 246,500 | 149,300 | 591,900 | 127,700 | 427,100 | 183,900 | 243,200 | 4,890 |
| ND | 55,100 | 17,500 | 4,800 | 32,800 | 5,900 | 24,000 | 7,700 | 16,300 | 4,100 |
| NE | 155,300 | 44,800 | 16,200 | 94,300 | 17,900 | 69,400 | 22,000 | 47,400 | 7,430 |
| NH | 114,200 | 33,700 | 9,800 | 70,700 | 16,500 | 48,100 | 17,900 | 30,200 | 4,440 |
| NJ | 854,500 | 235,300 | 93,500 | 525,700 | 145,000 | 333,500 | 152,100 | 181,400 | 4,450 |
| NM | 218,800 | 58,600 | 32,400 | 127,800 | 24,900 | 95,900 | 47,900 | 48,000 | 2,140 |
| NV | 251,200 | 75,100 | 35,000 | 141,100 | 29,100 | 102,800 | 47,100 | 55,700 | 7,700 |
| NY | 1,826,300 | 517,100 | 164,200 | 1,145,000 | 299,200 | 753,100 | 322,000 | 431,100 | 4,310 |
| OH | 1,157,500 | 285,600 | 138,100 | 733,800 | 154,700 | 519,100 | 219,600 | 299,500 | 6,660 |
| OK | 382,300 | 100,100 | 54,400 | 227,800 | 40,700 | 172,400 | 72,300 | 100,100 | 4,760 |
| OR | 343,900 | 98,100 | 39,900 | 205,900 | 47,800 | 140,500 | 60,100 | 80,400 | 2,470 |
| PA | 1,270,800 | 325,400 | 119,400 | 826,000 | 184,800 | 562,100 | 227,200 | 334,900 | 5,110 |
| RI | 92,400 | 27,200 | 10,100 | 55,100 | 12,800 | 36,700 | 15,700 | 21,000 | 5,960 |
| SC | 494,200 | 126,500 | 86,600 | 281,100 | 60,900 | 200,900 | 74,500 | 126,400 | 5,400 |
| SD | 71,900 | 20,500 | 7,200 | 44,200 | 9,300 | 32,100 | 11,500 | 20,600 | 6,750 |
| TN | 660,200 | 161,000 | 90,100 | 409,100 | 77,900 | 304,700 | 139,400 | 165,300 | 4,470 |
| TX | 2,564,400 | 663,200 | 436,400 | 1,464,800 | 275,300 | 1,097,300 | 580,700 | 516,600 | 5,730 |
| UT | 177,500 | 53,500 | 13,000 | 111,000 | 18,000 | 79,900 | 25,700 | 54,200 | 3,620 |
| VA | 765,600 | 206,800 | 77,600 | 481,200 | 91,600 | 341,500 | 149,100 | 192,400 | 6,640 |
| VT | 46,600 | 15,800 | 3,000 | 27,800 | 5,800 | 19,900 | 7,700 | 12,200 | 3,680 |
| WA | 605,900 | 172,600 | 53,100 | 380,200 | 71,500 | 276,000 | 120,000 | 156,000 | 2,790 |
| WI | 506,100 | 142,300 | 38,700 | 325,100 | 66,600 | 238,200 | 84,400 | 153,800 | 5,110 |
| WV | 237,300 | 48,400 | 35,400 | 153,500 | 31,100 | 111,100 | 46,400 | 64,700 | 4,500 |
| WY | 48,500 | 13,900 | 5,900 | 28,700 | 5,400 | 21,000 | 8,200 | 12,800 | 4,630 |
| **U.S.** | 30,207,400 | 8,129,300 | 3,739,900 | 18,338,200 | 3,900,000 | 12,990,000 | 5,550,000 | 7,440,000 | 4,860 |

Sources: ^a^ American Diabetes Association. Economic costs of diabetes in the US in 2012. *Diabetes care*. 2013; 36(4):1033-1046. ^b^ Dall TM, Yang W, Halder P et al. The economic burden of elevated blood glucose levels in 2012: diagnosed and undiagnosed diabetes, gestational diabetes mellitus, and prediabetes. *Diabetes care*. 2014;37(12):3172-3179.

Table B-2. Diabetes population percentage metrics by state, 2012

| **State** | **% Diabetes cases undiagnosed** | **Diagnosed diabetes** | | | |
| --- | --- | --- | --- | --- | --- |
|  |  | **% Uninsured** | **Insured** | | |
|  |  |  | **% Type 2 diabetes** | **Type 2 diabetes** | |
|  |  |  |  | **% Treated with Rx** | **Treated** |
|  |  |  |  |  | **% Controlled** |
| AK | 38 | 15 | 92 | 78 | 62 |
| AL | 23 | 19 | 92 | 80 | 59 |
| AR | 24 | 22 | 92 | 79 | 62 |
| AZ | 27 | 14 | 91 | 73 | 52 |
| CA | 30 | 18 | 92 | 80 | 62 |
| CO | 33 | 14 | 91 | 80 | 57 |
| CT | 30 | 11 | 91 | 75 | 58 |
| DC | 35 | 8 | 87 | 77 | 52 |
| DE | 25 | 9 | 91 | 75 | 56 |
| FL | 25 | 18 | 93 | 72 | 53 |
| GA | 25 | 20 | 91 | 78 | 55 |
| HI | 35 | 6 | 93 | 74 | 60 |
| IA | 29 | 11 | 93 | 80 | 71 |
| ID | 27 | 19 | 91 | 79 | 65 |
| IL | 29 | 20 | 91 | 79 | 57 |
| IN | 25 | 17 | 92 | 80 | 60 |
| KS | 26 | 15 | 92 | 80 | 62 |
| KY | 22 | 19 | 93 | 79 | 63 |
| LA | 25 | 26 | 93 | 80 | 58 |
| MA | 27 | 8 | 91 | 75 | 58 |
| MD | 28 | 11 | 92 | 75 | 51 |
| ME | 26 | 14 | 93 | 73 | 62 |
| MI | 26 | 16 | 92 | 74 | 58 |
| MN | 32 | 9 | 90 | 76 | 71 |
| MO | 27 | 16 | 91 | 78 | 54 |
| MS | 23 | 22 | 94 | 81 | 58 |
| MT | 35 | 20 | 93 | 78 | 61 |
| NC | 25 | 20 | 94 | 77 | 57 |
| ND | 32 | 13 | 91 | 80 | 68 |
| NE | 29 | 15 | 93 | 79 | 68 |
| NH | 30 | 12 | 91 | 74 | 63 |
| NJ | 28 | 15 | 91 | 70 | 54 |
| NM | 27 | 20 | 95 | 79 | 50 |
| NV | 30 | 20 | 93 | 78 | 54 |
| NY | 28 | 13 | 92 | 72 | 57 |
| OH | 25 | 16 | 92 | 77 | 58 |
| OK | 26 | 19 | 94 | 81 | 58 |
| OR | 29 | 16 | 91 | 75 | 57 |
| PA | 26 | 13 | 90 | 75 | 60 |
| RI | 29 | 15 | 90 | 74 | 57 |
| SC | 26 | 24 | 93 | 77 | 63 |
| SD | 29 | 14 | 94 | 78 | 64 |
| TN | 24 | 18 | 94 | 80 | 54 |
| TX | 26 | 23 | 94 | 80 | 47 |
| UT | 30 | 10 | 88 | 82 | 68 |
| VA | 27 | 14 | 90 | 79 | 56 |
| VT | 34 | 10 | 92 | 77 | 61 |
| WA | 28 | 12 | 91 | 79 | 57 |
| WI | 28 | 11 | 94 | 78 | 65 |
| WV | 20 | 19 | 93 | 78 | 58 |
| WY | 29 | 17 | 92 | 80 | 61 |
| **US** | **27** | **17** | **92** | **77** | **57** |

Table B-3: Commercially insured population with type 2 diabetes

|  | **Total type 2 population (thousands)** | | | | **Diagnosed population (thousands)** | | | **Treated population** | | | | | |
| --- | --- | --- | --- | --- | --- | --- | --- | --- | --- | --- | --- | --- | --- |
|  |  |  |  |  |  |  |  | **Controlled** | | **Uncontrolled** | | | |
| **State** | **Total** | **Diagnosed** | **Undiagnosed** | **% Undiagnosed** | **Treated** | **Not treated** | **% Not treated** | **Total (thousands)** | **Average annual medical expenditures ($)** | **Total (thousands)** | **Average annual medical expenditures ($)** | **Higher medical expenditures (vs controlled) ($)** | **% Uncontrolled** |
| AK | 18.8 | 11.2 | 7.6 | 40 | 9.1 | 2.1 | 19 | 5.2 | 12,260 | 3.9 | 18,810 | 6,550 | 43 |
| AL | 200.2 | 151.2 | 49.0 | 24 | 127.6 | 23.6 | 16 | 78.5 | 10,060 | 49.1 | 15,140 | 5,080 | 38 |
| AR | 99.7 | 74.2 | 25.5 | 26 | 60.6 | 13.6 | 18 | 34.9 | 12,460 | 25.7 | 19,230 | 6,770 | 42 |
| AZ | 204.4 | 148.3 | 56.1 | 27 | 121.8 | 26.5 | 18 | 59.5 | 8,600 | 62.3 | 15,670 | 7,070 | 51 |
| CA | 1,167.8 | 786.4 | 381.4 | 33 | 710.9 | 75.5 | 10 | 520.2 | 28,850 | 190.7 | 32,300 | 3,450 | 27 |
| CO | 131.3 | 81.6 | 49.7 | 38 | 68.7 | 12.9 | 16 | 37.7 | 11,850 | 31.0 | 18,090 | 6,240 | 45 |
| CT | 103.6 | 65.0 | 38.6 | 37 | 51.6 | 13.4 | 21 | 29.8 | 12,530 | 21.8 | 21,180 | 8,650 | 42 |
| DC | 16.8 | 9.9 | 6.9 | 41 | 8.4 | 1.5 | 15 | 4.7 | 11,460 | 3.7 | 17,610 | 6,150 | 44 |
| DE | 35.4 | 25.6 | 9.8 | 28 | 20.5 | 5.1 | 20 | 12.1 | 12,350 | 8.4 | 18,950 | 6,600 | 41 |
| FL | 643.5 | 469.8 | 173.7 | 27 | 365.7 | 104.1 | 22 | 194.6 | 11,650 | 171.1 | 17,960 | 6,310 | 47 |
| GA | 341.1 | 244.1 | 97.0 | 28 | 197.7 | 46.4 | 19 | 111.4 | 11,220 | 86.3 | 19,550 | 8,330 | 44 |
| HI | 53.1 | 32.7 | 20.4 | 38 | 26.6 | 6.1 | 19 | 15.2 | 12,320 | 11.4 | 18,700 | 6,380 | 43 |
| IA | 101.3 | 69.6 | 31.7 | 31 | 58.1 | 11.5 | 17 | 40.3 | 12,920 | 17.8 | 20,720 | 7,800 | 31 |
| ID | 44.8 | 31.4 | 13.4 | 30 | 25.5 | 5.9 | 19 | 15.7 | 13,140 | 9.8 | 19,160 | 6,020 | 38 |
| IL | 403.0 | 267.4 | 135.6 | 34 | 219.8 | 47.6 | 18 | 126.2 | 11,930 | 93.6 | 19,300 | 7,370 | 43 |
| IN | 228.1 | 166.1 | 62.0 | 27 | 136.8 | 29.3 | 18 | 84.7 | 13,780 | 52.1 | 22,110 | 8,330 | 38 |
| KS | 102.3 | 72.4 | 29.9 | 29 | 60.4 | 12.0 | 17 | 36.4 | 10,360 | 24.0 | 16,420 | 6,060 | 40 |
| KY | 168.6 | 127.1 | 41.5 | 25 | 103.0 | 24.1 | 19 | 68.1 | 11,890 | 34.9 | 18,560 | 6,670 | 34 |
| LA | 148.9 | 104.2 | 44.7 | 30 | 86.6 | 17.6 | 17 | 52.1 | 10,750 | 34.5 | 15,410 | 4,660 | 40 |
| MA | 219.9 | 149.7 | 70.2 | 32 | 114.8 | 34.9 | 23 | 65.0 | 10,280 | 49.8 | 19,430 | 9,150 | 43 |
| MD | 235.1 | 163.6 | 71.5 | 30 | 129.1 | 34.5 | 21 | 65.6 | 9,420 | 63.5 | 15,510 | 6,090 | 49 |
| ME | 42.3 | 30.0 | 12.3 | 29 | 22.3 | 7.7 | 26 | 13.8 | 12,780 | 8.5 | 19,380 | 6,600 | 38 |
| MI | 341.1 | 241.1 | 100.0 | 29 | 192.3 | 48.8 | 20 | 113.7 | 11,220 | 78.6 | 17,800 | 6,580 | 41 |
| MN | 144.1 | 88.0 | 56.1 | 39 | 68.4 | 19.6 | 22 | 49.9 | 13,070 | 18.5 | 22,240 | 9,170 | 27 |
| MO | 199.1 | 140.9 | 58.2 | 29 | 115.6 | 25.3 | 18 | 59.8 | 10,210 | 55.8 | 17,730 | 7,520 | 48 |
| MS | 105.4 | 79.1 | 26.3 | 25 | 67.4 | 11.7 | 15 | 40.2 | 11,500 | 27.2 | 15,980 | 4,480 | 40 |
| MT | 22.3 | 13.2 | 9.1 | 41 | 10.7 | 2.5 | 19 | 6.1 | 12,560 | 4.6 | 18,940 | 6,380 | 43 |
| NC | 318.6 | 227.2 | 91.4 | 29 | 183.8 | 43.4 | 19 | 107.5 | 12,380 | 76.3 | 18,800 | 6,420 | 42 |
| ND | 20.5 | 12.6 | 7.9 | 39 | 10.6 | 2.0 | 16 | 6.5 | 12,440 | 4.1 | 19,110 | 6,670 | 39 |
| NE | 57.2 | 37.8 | 19.4 | 34 | 30.8 | 7.0 | 19 | 19.9 | 11,340 | 10.9 | 21,000 | 9,660 | 35 |
| NH | 42.7 | 28.2 | 14.5 | 34 | 22.1 | 6.1 | 22 | 13.5 | 13,120 | 8.6 | 18,640 | 5,520 | 39 |
| NJ | 308.4 | 209.9 | 98.5 | 32 | 159.6 | 50.3 | 24 | 90.3 | 12,080 | 69.3 | 17,980 | 5,900 | 43 |
| NM | 66.6 | 47.4 | 19.2 | 29 | 39.3 | 8.1 | 17 | 20.7 | 9,010 | 18.6 | 14,150 | 5,140 | 47 |
| NV | 86.7 | 59.3 | 27.4 | 32 | 48.4 | 10.9 | 18 | 26.7 | 11,160 | 21.7 | 20,830 | 9,670 | 45 |
| NY | 593.2 | 397.9 | 195.3 | 33 | 313.7 | 84.2 | 21 | 184.6 | 12,400 | 129.1 | 18,700 | 6,300 | 41 |
| OH | 422.9 | 311.3 | 111.6 | 26 | 250.1 | 61.2 | 20 | 141.0 | 12,230 | 109.1 | 19,570 | 7,340 | 44 |
| OK | 122.8 | 87.5 | 35.3 | 29 | 73.8 | 13.7 | 16 | 43.8 | 14,610 | 30.0 | 21,640 | 7,030 | 41 |
| OR | 113.8 | 78.6 | 35.2 | 31 | 62.4 | 16.2 | 21 | 35.7 | 13,130 | 26.7 | 17,650 | 4,520 | 43 |
| PA | 431.6 | 300.0 | 131.6 | 30 | 238.8 | 61.2 | 20 | 140.4 | 12,530 | 98.4 | 20,780 | 8,250 | 41 |
| RI | 31.6 | 21.0 | 10.6 | 34 | 16.4 | 4.6 | 22 | 9.4 | 12,680 | 7.0 | 19,580 | 6,900 | 43 |
| SC | 151.5 | 107.6 | 43.9 | 29 | 86.2 | 21.4 | 20 | 55.3 | 12,270 | 30.9 | 19,940 | 7,670 | 36 |
| SD | 22.9 | 14.6 | 8.3 | 36 | 11.8 | 2.8 | 19 | 6.8 | 13,070 | 5.0 | 19,820 | 6,750 | 42 |
| TN | 232.0 | 171.8 | 60.2 | 26 | 142.1 | 29.7 | 17 | 76.8 | 10,460 | 65.3 | 17,110 | 6,650 | 46 |
| TX | 887.0 | 641.6 | 245.4 | 28 | 544.4 | 97.2 | 15 | 254.5 | 11,650 | 289.9 | 18,700 | 7,050 | 53 |
| UT | 69.1 | 45.1 | 24.0 | 35 | 39.4 | 5.7 | 13 | 26.6 | 12,110 | 12.8 | 19,330 | 7,220 | 32 |
| VA | 298.6 | 206.5 | 92.1 | 31 | 173.0 | 33.5 | 16 | 97.1 | 10,530 | 75.9 | 20,740 | 10,210 | 44 |
| VT | 15.7 | 9.7 | 6.0 | 38 | 7.9 | 1.8 | 19 | 4.6 | 13,020 | 3.3 | 20,110 | 7,090 | 42 |
| WA | 238.9 | 166.5 | 72.4 | 30 | 137.4 | 29.1 | 17 | 77.7 | 14,520 | 59.7 | 18,260 | 3,740 | 43 |
| WI | 186.0 | 126.4 | 59.6 | 32 | 103.8 | 22.6 | 18 | 67.2 | 12,870 | 36.6 | 21,270 | 8,400 | 35 |
| WV | 73.3 | 56.8 | 16.5 | 23 | 45.4 | 11.4 | 20 | 27.0 | 14,200 | 18.4 | 18,930 | 4,730 | 41 |
| WY | 16.4 | 10.9 | 5.5 | 34 | 8.8 | 2.1 | 19 | 5.0 | 12,730 | 3.8 | 18,810 | 6,080 | 43 |
| **US** | **10,330** | **7,220** | **3,110** | **30** | **5,930** | **1,290** | **18** | **3,480** | **13,050** | **2,450** | **19,730** | **6,680** | **41** |

Table B-4: Medicaid population with type 2 diabetes

|  | **Total type 2 population (thousands)** | | | | **Diagnosed population (thousands)** | | | **Treated population** | | | | | |
| --- | --- | --- | --- | --- | --- | --- | --- | --- | --- | --- | --- | --- | --- |
|  |  |  |  |  |  |  |  | **Controlled** | | **Uncontrolled** | | | |
| **State** | **Total** | **Diagnosed** | **Undiagnosed** | **% Undiagnosed** | **Treated** | **Not treated** | **% Not treated** | **Total (thousands)** | **Average annual medical expenditures ($)** | **Total (thousands)** | **Average annual medical expenditures ($)** | **Higher medical expenditures (vs controlled) ($)** | **% Uncontrolled** |
| AK | 5.9 | 2.2 | 3.7 | 63 | 1.7 | 0.5 | 23 | 1.0 | 19,210 | 0.7 | 23,730 | 4,520 | 41 |
| AL | 33.4 | 25.3 | 8.1 | 24 | 19.5 | 5.8 | 23 | 9.7 | 13,330 | 9.8 | 14,820 | 1,490 | 50 |
| AR | 20.7 | 15.5 | 5.2 | 25 | 11.9 | 3.6 | 23 | 7.9 | 17,310 | 4.0 | 21,770 | 4,460 | 34 |
| AZ | 78.6 | 59.4 | 19.2 | 24 | 26.2 | 33.2 | 56 | 15.6 | 20,710 | 10.6 | 23,870 | 3,160 | 40 |
| CA | 311.3 | 212.5 | 98.8 | 32 | 156.3 | 56.2 | 26 | 94.1 | 16,410 | 62.2 | 18,860 | 2,450 | 40 |
| CO | 25.1 | 16.3 | 8.8 | 35 | 12.8 | 3.5 | 21 | 6.9 | 22,210 | 5.9 | 27,350 | 5,140 | 46 |
| CT | 27.9 | 20.0 | 7.9 | 28 | 16.8 | 3.2 | 16 | 11.2 | 26,640 | 5.6 | 32,150 | 5,510 | 33 |
| DC | 8.2 | 4.8 | 3.4 | 41 | 3.5 | 1.3 | 27 | 1.6 | 21,720 | 1.9 | 34,590 | 12,870 | 54 |
| DE | 8.6 | 6.0 | 2.6 | 30 | 4.5 | 1.5 | 25 | 2.5 | 21,100 | 2.0 | 24,870 | 3,770 | 44 |
| FL | 131.5 | 92.5 | 39.0 | 30 | 67.9 | 24.6 | 27 | 37.4 | 16,290 | 30.5 | 21,510 | 5,220 | 45 |
| GA | 53.6 | 39.0 | 14.6 | 27 | 30.8 | 8.2 | 21 | 16.2 | 17,690 | 14.6 | 22,550 | 4,860 | 47 |
| HI | 8.5 | 4.4 | 4.1 | 48 | 2.9 | 1.5 | 34 | 1.4 | 19,120 | 1.5 | 20,450 | 1,330 | 52 |
| IA | 15.5 | 10.8 | 4.7 | 30 | 8.9 | 1.9 | 18 | 5.5 | 23,960 | 3.4 | 26,950 | 2,990 | 38 |
| ID | 6.8 | 5.0 | 1.8 | 26 | 4.1 | 0.9 | 18 | 2.5 | 21,450 | 1.6 | 24,280 | 2,830 | 39 |
| IL | 83.6 | 57.8 | 25.8 | 31 | 46.0 | 11.8 | 20 | 28.3 | 18,590 | 17.7 | 23,750 | 5,160 | 38 |
| IN | 36.0 | 26.4 | 9.6 | 27 | 20.6 | 5.8 | 22 | 10.9 | 20,960 | 9.7 | 23,990 | 3,030 | 47 |
| KS | 9.3 | 6.4 | 2.9 | 31 | 5.2 | 1.2 | 19 | 3.0 | 24,660 | 2.2 | 31,680 | 7,020 | 42 |
| KY | 37.3 | 28.7 | 8.6 | 23 | 22.0 | 6.7 | 23 | 12.8 | 19,160 | 9.2 | 27,890 | 8,730 | 42 |
| LA | 37.3 | 27.2 | 10.1 | 27 | 22.0 | 5.2 | 19 | 13.1 | 21,300 | 8.9 | 24,510 | 3,210 | 40 |
| MA | 77.5 | 53.5 | 24.0 | 31 | 43.3 | 10.2 | 19 | 27.2 | 20,060 | 16.1 | 27,320 | 7,260 | 37 |
| MD | 36.7 | 25.5 | 11.2 | 31 | 20.2 | 5.3 | 21 | 9.4 | 25,320 | 10.8 | 30,220 | 4,900 | 53 |
| ME | 12.8 | 8.7 | 4.1 | 32 | 6.8 | 1.9 | 22 | 3.9 | 19,120 | 2.9 | 23,650 | 4,530 | 43 |
| MI | 85.7 | 63.4 | 22.3 | 26 | 42.8 | 20.6 | 32 | 25.2 | 14,270 | 17.6 | 16,180 | 1,910 | 41 |
| MN | 35.1 | 24.2 | 10.9 | 31 | 18.7 | 5.5 | 23 | 11.2 | 27,400 | 7.5 | 27,640 | 240 | 40 |
| MO | 32.2 | 22.5 | 9.7 | 30 | 18.3 | 4.2 | 19 | 10.5 | 21,500 | 7.8 | 22,980 | 1,480 | 43 |
| MS | 28.3 | 20.8 | 7.5 | 27 | 15.3 | 5.5 | 26 | 8.4 | 15,390 | 6.9 | 19,300 | 3,910 | 45 |
| MT | 6.1 | 3.4 | 2.7 | 44 | 2.5 | 0.9 | 26 | 1.6 | 19,620 | 0.9 | 23,400 | 3,780 | 36 |
| NC | 58.4 | 42.0 | 16.4 | 28 | 33.9 | 8.1 | 19 | 15.2 | 18,700 | 18.7 | 25,840 | 7,140 | 55 |
| ND | 2.8 | 1.7 | 1.1 | 39 | 1.4 | 0.3 | 18 | 0.9 | 19,210 | 0.5 | 23,810 | 4,600 | 36 |
| NE | 6.3 | 4.4 | 1.9 | 30 | 3.6 | 0.8 | 18 | 2.3 | 18,870 | 1.3 | 23,370 | 4,500 | 36 |
| NH | 3.7 | 2.5 | 1.2 | 32 | 1.9 | 0.6 | 24 | 1.1 | 20,670 | 0.8 | 23,940 | 3,270 | 42 |
| NJ | 40.8 | 26.3 | 14.5 | 36 | 19.3 | 7.0 | 27 | 11.6 | 24,240 | 7.7 | 29,710 | 5,470 | 40 |
| NM | 23.1 | 15.0 | 8.1 | 35 | 12.2 | 2.8 | 19 | 6.6 | 22,090 | 5.6 | 24,040 | 1,950 | 46 |
| NV | 10.2 | 6.3 | 3.9 | 38 | 4.9 | 1.4 | 22 | 2.7 | 19,450 | 2.2 | 24,750 | 5,300 | 45 |
| NY | 223.1 | 153.7 | 69.4 | 31 | 125.6 | 28.1 | 18 | 78.3 | 21,510 | 47.3 | 27,800 | 6,290 | 38 |
| OH | 86.3 | 64.1 | 22.2 | 26 | 46.5 | 17.6 | 27 | 31.1 | 26,640 | 15.4 | 40,400 | 13,760 | 33 |
| OK | 34.0 | 23.3 | 10.7 | 31 | 18.7 | 4.6 | 20 | 9.6 | 20,100 | 9.1 | 25,550 | 5,450 | 49 |
| OR | 25.0 | 17.5 | 7.5 | 30 | 10.2 | 7.3 | 42 | 5.7 | 21,580 | 4.5 | 24,230 | 2,650 | 44 |
| PA | 83.0 | 59.1 | 23.9 | 29 | 45.1 | 14.0 | 24 | 28.4 | 22,780 | 16.7 | 24,310 | 1,530 | 37 |
| RI | 6.3 | 4.2 | 2.1 | 33 | 3.4 | 0.8 | 19 | 1.9 | 19,170 | 1.5 | 24,050 | 4,880 | 44 |
| SC | 31.5 | 22.6 | 8.9 | 28 | 17.6 | 5.0 | 22 | 10.1 | 16,900 | 7.5 | 22,700 | 5,800 | 43 |
| SD | 6.5 | 4.6 | 1.9 | 29 | 3.0 | 1.6 | 35 | 1.8 | 17,920 | 1.2 | 22,170 | 4,250 | 40 |
| TN | 47.0 | 34.3 | 12.7 | 27 | 27.6 | 6.7 | 20 | 14.4 | 13,820 | 13.2 | 17,360 | 3,540 | 48 |
| TX | 157.9 | 116.3 | 41.6 | 26 | 86.9 | 29.4 | 25 | 39.4 | 17,450 | 47.5 | 19,670 | 2,220 | 55 |
| UT | 10.0 | 7.4 | 2.6 | 26 | 5.8 | 1.6 | 22 | 3.8 | 19,000 | 2.0 | 22,980 | 3,980 | 34 |
| VA | 34.9 | 26.9 | 8.0 | 23 | 19.7 | 7.2 | 27 | 11.3 | 22,410 | 8.4 | 23,950 | 1,540 | 43 |
| VT | 5.3 | 3.3 | 2.0 | 38 | 2.6 | 0.7 | 21 | 1.4 | 18,810 | 1.2 | 23,370 | 4,560 | 46 |
| WA | 40.8 | 29.9 | 10.9 | 27 | 24.8 | 5.1 | 17 | 14.3 | 16,860 | 10.5 | 21,660 | 4,800 | 42 |
| WI | 36.3 | 23.9 | 12.4 | 34 | 19.0 | 4.9 | 21 | 11.0 | 15,570 | 8.0 | 19,480 | 3,910 | 42 |
| WV | 21.4 | 17.4 | 4.0 | 19 | 13.9 | 3.5 | 20 | 7.5 | 15,580 | 6.4 | 20,860 | 5,280 | 46 |
| WY | 1.9 | 1.1 | 0.8 | 42 | 0.9 | 0.2 | 18 | 0.6 | 23,530 | 0.3 | 28,190 | 4,660 | 33 |
| **US** | **2,250** | **1,590** | **660** | **29** | **1,200** | **390** | **25** | **690** | **19,130** | **510** | **23,490** | **4,360** | **43** |

Table B-5: Medicare population with type 2 diabetes

|  | **Total type 2 population (thousands)** | | | | **Diagnosed population (thousands)** | | | **Treated population** | | | | | |
| --- | --- | --- | --- | --- | --- | --- | --- | --- | --- | --- | --- | --- | --- |
|  |  |  |  |  |  |  |  | **Controlled** | | **Uncontrolled** | | | |
| **State** | **Total** | **Diagnosed** | **Undiagnosed** | **% Undiagnosed** | **Treated** | **Not treated** | **% Not treated** | **Total (thousands)** | **Average annual medical expenditures ($)** | **Total (thousands)** | **Average annual medical expenditures ($)** | **Higher medical expenditures (vs controlled) ($)** | **% Uncontrolled** |
| AK | 15.5 | 10.3 | 5.2 | 34 | 7.8 | 2.5 | 24 | 5.3 | 18,570 | 2.5 | 21,160 | 2,590 | 32 |
| AL | 186.4 | 137.6 | 48.8 | 26 | 103.3 | 34.3 | 25 | 58.8 | 19,340 | 44.5 | 21,950 | 2,610 | 43 |
| AR | 111.8 | 80.8 | 31.0 | 28 | 62.6 | 18.2 | 23 | 40.6 | 18,920 | 22.0 | 21,930 | 3,010 | 35 |
| AZ | 230.2 | 158.6 | 71.6 | 31 | 118.1 | 40.5 | 26 | 62.6 | 15,560 | 55.5 | 22,480 | 6,920 | 47 |
| CA | 1,172.6 | 816.9 | 355.7 | 30 | 586.4 | 230.5 | 28 | 291.6 | 23,180 | 294.8 | 25,640 | 2,460 | 50 |
| CO | 128.9 | 86.3 | 42.6 | 33 | 66.6 | 19.7 | 23 | 39.5 | 16,670 | 27.1 | 23,320 | 6,650 | 41 |
| CT | 125.1 | 87.8 | 37.3 | 30 | 60.8 | 27.0 | 31 | 34.4 | 23,640 | 26.4 | 28,030 | 4,390 | 43 |
| DC | 16.8 | 10.6 | 6.2 | 37 | 7.7 | 2.9 | 27 | 3.8 | 19,440 | 3.9 | 25,020 | 5,580 | 51 |
| DE | 39.5 | 28.9 | 10.6 | 27 | 20.5 | 8.4 | 29 | 10.9 | 18,540 | 9.6 | 24,720 | 6,180 | 47 |
| FL | 1,013.3 | 749.9 | 263.4 | 26 | 507.9 | 242.0 | 32 | 264.1 | 25,240 | 243.8 | 27,470 | 2,230 | 48 |
| GA | 313.9 | 233.9 | 80.0 | 25 | 173.6 | 60.3 | 26 | 94.5 | 18,760 | 79.1 | 21,250 | 2,490 | 46 |
| HI | 55.7 | 36.3 | 19.4 | 35 | 24.6 | 11.7 | 32 | 15.7 | 20,150 | 8.9 | 19,030 | (1,120) | 36 |
| IA | 105.0 | 71.9 | 33.1 | 32 | 54.8 | 17.1 | 24 | 40.1 | 17,830 | 14.7 | 21,870 | 4,040 | 27 |
| ID | 50.6 | 36.2 | 14.4 | 28 | 27.7 | 8.5 | 23 | 18.8 | 16,790 | 8.9 | 21,490 | 4,700 | 32 |
| IL | 413.7 | 282.7 | 131.0 | 32 | 213.1 | 69.6 | 25 | 120.0 | 20,270 | 93.1 | 24,630 | 4,360 | 44 |
| IN | 238.4 | 173.1 | 65.3 | 27 | 133.9 | 39.2 | 23 | 78.9 | 18,710 | 55.0 | 22,940 | 4,230 | 41 |
| KS | 102.0 | 74.9 | 27.1 | 27 | 56.9 | 18.0 | 24 | 36.6 | 17,630 | 20.3 | 22,140 | 4,510 | 36 |
| KY | 172.4 | 130.9 | 41.5 | 24 | 100.3 | 30.6 | 23 | 60.6 | 19,420 | 39.7 | 23,130 | 3,710 | 40 |
| LA | 174.6 | 129.5 | 45.1 | 26 | 100.1 | 29.4 | 23 | 56.2 | 23,340 | 43.9 | 27,470 | 4,130 | 44 |
| MA | 232.3 | 167.6 | 64.7 | 28 | 118.8 | 48.8 | 29 | 67.6 | 21,290 | 51.2 | 25,990 | 4,700 | 43 |
| MD | 202.2 | 145.8 | 56.4 | 28 | 102.8 | 43.0 | 29 | 52.8 | 21,920 | 50.0 | 27,470 | 5,550 | 49 |
| ME | 56.7 | 41.0 | 15.7 | 28 | 29.3 | 11.7 | 29 | 18.7 | 19,700 | 10.6 | 20,890 | 1,190 | 36 |
| MI | 374.9 | 270.1 | 104.8 | 28 | 189.0 | 81.1 | 30 | 108.9 | 20,900 | 80.1 | 24,990 | 4,090 | 42 |
| MN | 163.0 | 112.9 | 50.1 | 31 | 85.0 | 27.9 | 25 | 60.5 | 16,480 | 24.5 | 19,660 | 3,180 | 29 |
| MO | 215.4 | 152.8 | 62.6 | 29 | 114.0 | 38.8 | 25 | 64.3 | 18,700 | 49.7 | 21,100 | 2,400 | 44 |
| MS | 118.5 | 89.3 | 29.2 | 25 | 70.4 | 18.9 | 21 | 39.9 | 19,530 | 30.5 | 20,830 | 1,300 | 43 |
| MT | 31.0 | 19.7 | 11.3 | 36 | 15.2 | 4.5 | 23 | 9.7 | 14,990 | 5.5 | 19,400 | 4,410 | 36 |
| NC | 379.2 | 285.6 | 93.6 | 25 | 209.4 | 76.2 | 27 | 120.5 | 18,220 | 88.9 | 21,450 | 3,230 | 42 |
| ND | 22.7 | 15.6 | 7.1 | 31 | 12.0 | 3.6 | 23 | 8.9 | 17,250 | 3.1 | 19,350 | 2,100 | 26 |
| NE | 63.6 | 45.1 | 18.5 | 29 | 35.0 | 10.1 | 22 | 25.2 | 17,620 | 9.8 | 23,720 | 6,100 | 28 |
| NH | 48.0 | 33.9 | 14.1 | 29 | 24.1 | 9.8 | 29 | 15.6 | 18,810 | 8.5 | 22,580 | 3,770 | 35 |
| NJ | 333.9 | 242.3 | 91.6 | 27 | 154.6 | 87.7 | 36 | 79.5 | 24,430 | 75.1 | 26,810 | 2,380 | 49 |
| NM | 80.9 | 58.4 | 22.5 | 28 | 44.4 | 14.0 | 24 | 20.7 | 16,070 | 23.7 | 16,180 | 110 | 53 |
| NV | 95.3 | 66.3 | 29.0 | 30 | 49.5 | 16.8 | 25 | 26.3 | 22,440 | 23.2 | 28,100 | 5,660 | 47 |
| NY | 703.9 | 500.7 | 203.2 | 29 | 313.8 | 186.9 | 37 | 168.2 | 26,990 | 145.6 | 28,520 | 1,530 | 46 |
| OH | 417.4 | 298.4 | 119.0 | 29 | 222.5 | 75.9 | 25 | 127.4 | 20,530 | 95.1 | 25,730 | 5,200 | 43 |
| OK | 141.5 | 102.3 | 39.2 | 28 | 79.9 | 22.4 | 22 | 46.7 | 19,240 | 33.2 | 22,510 | 3,270 | 42 |
| OR | 133.4 | 92.2 | 41.2 | 31 | 67.9 | 24.3 | 26 | 39.0 | 16,590 | 28.9 | 17,780 | 1,190 | 43 |
| PA | 527.3 | 387.8 | 139.5 | 26 | 278.2 | 109.6 | 28 | 166.1 | 22,070 | 112.1 | 24,960 | 2,890 | 40 |
| RI | 35.7 | 24.3 | 11.4 | 32 | 16.9 | 7.4 | 30 | 9.7 | 18,090 | 7.2 | 23,870 | 5,780 | 43 |
| SC | 182.5 | 131.6 | 50.9 | 28 | 97.1 | 34.5 | 26 | 61.0 | 17,720 | 36.1 | 21,290 | 3,570 | 37 |
| SD | 30.9 | 22.2 | 8.7 | 28 | 17.3 | 4.9 | 22 | 12.0 | 15,290 | 5.3 | 23,340 | 8,050 | 31 |
| TN | 239.6 | 176.5 | 63.1 | 26 | 135.0 | 41.5 | 24 | 74.1 | 19,180 | 60.9 | 21,740 | 2,560 | 45 |
| TX | 834.6 | 614.7 | 219.9 | 26 | 466.0 | 148.7 | 24 | 222.7 | 20,700 | 243.3 | 26,110 | 5,410 | 52 |
| UT | 64.2 | 45.4 | 18.8 | 29 | 34.7 | 10.7 | 24 | 23.8 | 20,910 | 10.9 | 21,040 | 130 | 31 |
| VA | 277.0 | 199.7 | 77.3 | 28 | 148.8 | 50.9 | 25 | 84.0 | 16,520 | 64.8 | 20,800 | 4,280 | 44 |
| VT | 19.9 | 12.7 | 7.2 | 36 | 9.4 | 3.3 | 26 | 6.2 | 20,930 | 3.2 | 21,760 | 830 | 34 |
| WA | 217.4 | 151.1 | 66.3 | 30 | 113.8 | 37.3 | 25 | 64.0 | 16,970 | 49.8 | 19,480 | 2,510 | 44 |
| WI | 212.2 | 154.5 | 57.7 | 27 | 115.4 | 39.1 | 25 | 75.6 | 17,310 | 39.8 | 20,240 | 2,930 | 34 |
| WV | 88.6 | 68.0 | 20.6 | 23 | 51.8 | 16.2 | 24 | 30.2 | 20,630 | 21.6 | 24,730 | 4,100 | 42 |
| WY | 19.9 | 14.4 | 5.5 | 28 | 11.3 | 3.1 | 22 | 7.2 | 17,340 | 4.1 | 20,860 | 3,520 | 36 |
| **US** | **11,230** | **8,080** | **3,150** | **28** | **5,860** | **2,220** | **27** | **3,270** | **21,010** | **2,590** | **24,440** | **3,430** | **44** |

Table B-6: Total insured population with type 2 diabetes

|  | **Total type 2 population (thousands)** | | | | **Diagnosed population (thousands)** | | | **Treated population** | | | | | |
| --- | --- | --- | --- | --- | --- | --- | --- | --- | --- | --- | --- | --- | --- |
|  |  |  |  |  |  |  |  | **Controlled** | | **Uncontrolled** | | | |
| **State** | **Total** | **Diagnosed** | **Undiagnosed** | **% Undiagnosed** | **Treated** | **Not treated** | **% Not treated** | **Total (thousands)** | **Average annual medical expenditures ($)** | **Total (thousands)** | **Average annual medical expenditures ($)** | **Higher medical expenditures (vs controlled) ($)** | **% Uncontrolled** |
| AK | 45 | 24 | 18 | 41 | 19 | 5 | 22 | 12 | 15,780 | 7 | 19,990 | 4,210 | 38 |
| AL | 500 | 314 | 127 | 25 | 250 | 64 | 20 | 147 | 14,460 | 103 | 18,080 | 3,620 | 41 |
| AR | 285 | 171 | 75 | 26 | 135 | 35 | 21 | 83 | 15,830 | 52 | 20,460 | 4,630 | 38 |
| AZ | 578 | 366 | 172 | 30 | 266 | 100 | 27 | 138 | 12,970 | 128 | 19,420 | 6,450 | 48 |
| CA | 3,090 | 1,816 | 1,021 | 33 | 1,454 | 362 | 20 | 906 | 24,720 | 548 | 27,210 | 2,490 | 38 |
| CO | 320 | 184 | 118 | 37 | 148 | 36 | 20 | 84 | 15,400 | 64 | 21,290 | 5,890 | 43 |
| CT | 281 | 173 | 93 | 33 | 129 | 44 | 25 | 75 | 19,850 | 54 | 26,150 | 6,300 | 42 |
| DC | 44 | 25 | 17 | 38 | 20 | 6 | 23 | 10 | 18,030 | 10 | 25,190 | 7,160 | 48 |
| DE | 91 | 61 | 25 | 28 | 46 | 15 | 25 | 26 | 16,590 | 20 | 22,590 | 6,000 | 44 |
| FL | 2,099 | 1,312 | 579 | 28 | 942 | 371 | 28 | 496 | 18,980 | 445 | 23,720 | 4,740 | 47 |
| GA | 853 | 517 | 241 | 28 | 402 | 115 | 22 | 222 | 15,270 | 180 | 20,470 | 5,200 | 45 |
| HI | 123 | 73 | 46 | 38 | 54 | 19 | 26 | 32 | 16,220 | 22 | 18,710 | 2,490 | 40 |
| IA | 242 | 152 | 75 | 31 | 122 | 31 | 20 | 86 | 16,640 | 36 | 21,760 | 5,120 | 29 |
| ID | 121 | 73 | 36 | 30 | 57 | 15 | 21 | 37 | 15,840 | 20 | 20,550 | 4,710 | 35 |
| IL | 1,063 | 608 | 341 | 32 | 479 | 129 | 21 | 275 | 16,500 | 204 | 22,210 | 5,710 | 43 |
| IN | 584 | 366 | 160 | 27 | 291 | 74 | 20 | 175 | 17,030 | 117 | 22,640 | 5,610 | 40 |
| KS | 243 | 154 | 69 | 28 | 123 | 31 | 20 | 76 | 14,370 | 47 | 19,600 | 5,230 | 38 |
| KY | 450 | 287 | 108 | 24 | 225 | 61 | 21 | 142 | 16,600 | 84 | 22,120 | 5,520 | 37 |
| LA | 457 | 261 | 124 | 27 | 209 | 52 | 20 | 121 | 18,280 | 87 | 22,930 | 4,650 | 42 |
| MA | 564 | 371 | 162 | 29 | 277 | 94 | 25 | 160 | 16,980 | 117 | 23,860 | 6,880 | 42 |
| MD | 517 | 335 | 156 | 30 | 252 | 83 | 25 | 128 | 16,230 | 124 | 21,870 | 5,640 | 49 |
| ME | 126 | 80 | 36 | 28 | 58 | 21 | 27 | 36 | 17,430 | 22 | 21,040 | 3,610 | 38 |
| MI | 923 | 575 | 259 | 28 | 424 | 151 | 26 | 248 | 16,000 | 176 | 21,010 | 5,010 | 42 |
| MN | 367 | 225 | 126 | 34 | 172 | 53 | 24 | 122 | 18,010 | 51 | 22,260 | 4,250 | 29 |
| MO | 515 | 316 | 152 | 30 | 248 | 68 | 22 | 135 | 14,920 | 113 | 19,500 | 4,580 | 46 |
| MS | 311 | 189 | 79 | 25 | 153 | 36 | 19 | 89 | 15,870 | 65 | 18,850 | 2,980 | 42 |
| MT | 69 | 36 | 26 | 38 | 28 | 8 | 22 | 17 | 14,680 | 11 | 19,620 | 4,940 | 39 |
| NC | 905 | 555 | 247 | 27 | 427 | 128 | 23 | 243 | 16,160 | 184 | 21,050 | 4,890 | 43 |
| ND | 51 | 30 | 18 | 34 | 24 | 6 | 20 | 16 | 15,150 | 8 | 19,250 | 4,100 | 32 |
| NE | 143 | 87 | 45 | 31 | 69 | 18 | 21 | 47 | 14,730 | 22 | 22,160 | 7,430 | 32 |
| NH | 104 | 65 | 34 | 32 | 48 | 17 | 26 | 30 | 16,190 | 18 | 20,630 | 4,440 | 37 |
| NJ | 777 | 479 | 235 | 30 | 334 | 145 | 30 | 181 | 18,620 | 152 | 23,070 | 4,450 | 46 |
| NM | 203 | 121 | 59 | 29 | 96 | 25 | 21 | 48 | 14,560 | 48 | 16,700 | 2,140 | 50 |
| NV | 227 | 132 | 75 | 33 | 103 | 29 | 22 | 56 | 16,870 | 47 | 24,570 | 7,700 | 46 |
| NY | 1,684 | 1,052 | 517 | 31 | 753 | 299 | 28 | 431 | 20,830 | 322 | 25,140 | 4,310 | 43 |
| OH | 1,065 | 674 | 286 | 27 | 519 | 155 | 23 | 300 | 17,200 | 220 | 23,860 | 6,660 | 42 |
| OK | 353 | 213 | 100 | 28 | 172 | 41 | 19 | 100 | 18,040 | 72 | 22,800 | 4,760 | 42 |
| OR | 312 | 188 | 98 | 31 | 141 | 48 | 25 | 80 | 15,760 | 60 | 18,230 | 2,470 | 43 |
| PA | 1,161 | 747 | 325 | 28 | 562 | 185 | 25 | 335 | 18,070 | 227 | 23,180 | 5,110 | 40 |
| RI | 84 | 50 | 27 | 32 | 37 | 13 | 26 | 21 | 16,150 | 16 | 22,110 | 5,960 | 43 |
| SC | 452 | 262 | 127 | 28 | 201 | 61 | 23 | 126 | 15,630 | 75 | 21,030 | 5,400 | 37 |
| SD | 68 | 41 | 21 | 30 | 32 | 9 | 22 | 21 | 15,090 | 12 | 21,840 | 6,750 | 36 |
| TN | 609 | 383 | 161 | 26 | 305 | 78 | 20 | 165 | 14,710 | 139 | 19,180 | 4,470 | 46 |
| TX | 2,316 | 1,373 | 663 | 29 | 1,097 | 275 | 20 | 517 | 16,120 | 581 | 21,850 | 5,730 | 53 |
| UT | 156 | 98 | 54 | 34 | 80 | 18 | 18 | 54 | 16,600 | 26 | 20,220 | 3,620 | 32 |
| VA | 688 | 433 | 207 | 30 | 342 | 92 | 21 | 192 | 14,030 | 149 | 20,670 | 6,640 | 44 |
| VT | 44 | 26 | 16 | 36 | 20 | 6 | 23 | 12 | 17,950 | 8 | 21,630 | 3,680 | 39 |
| WA | 550 | 348 | 173 | 31 | 276 | 72 | 21 | 156 | 16,190 | 120 | 18,980 | 2,790 | 43 |
| WI | 473 | 305 | 142 | 30 | 238 | 67 | 22 | 154 | 15,460 | 84 | 20,570 | 5,110 | 35 |
| WV | 219 | 142 | 48 | 22 | 111 | 31 | 22 | 65 | 17,770 | 46 | 22,270 | 4,500 | 42 |
| WY | 44 | 26 | 14 | 32 | 21 | 5 | 20 | 13 | 15,430 | 8 | 20,060 | 4,630 | 39 |
| **US** | **27,546** | **16,890** | **8,129** | **30** | **12,990** | **3,900** | **23** | **7,440** | **17,550** | **5,550** | **22,410** | **4,860** | **43** |

Reference list

(1) American Diabetes Association. Standards of Medical Care in Diabetes−2015. *Diabetes care* 2015;38(Supplement 1):S1-S93.

(2) Centers for Disease Control and Prevention (CDC). National Diabetes Statistics Report, 2014. *Centers for Disease Control and Prevention* 2014;Available at: URL: http://www.cdc.gov/diabetes/pubs/statsreport14/national-diabetes-report-web.pdf.

(3) American Diabetes Association. Economic costs of diabetes in the U.S. in 2012. *Diabetes care* 2013.

(4) Dall TM, Yang W, Halder P et al. The economic burden of elevated blood glucose levels in 2012: diagnosed and undiagnosed diabetes, gestational diabetes mellitus, and prediabetes. *Diabetes care* 2014;37(12):3172-3179.

(5) Zhang Y, Dall TM, Mann SE et al. The economic costs of undiagnosed diabetes. *Population Health Management* 2009;12(2):95-101.

(6) Fitch K, Pyenson BS, Iwasaki K. Medical claim cost impact of improved diabetes control for medicare and commercially insured patients with type 2 diabetes. *J Manag Care Pharm* 2013;19(8):609-620d.

(7) Hill SC, Miller GE, Sing M. Adults with diagnosed and untreated diabetes: who are they? How can we reach them? *J Health Care Poor Underserved* 2011;22(4):1221-1238.

(8) Li R, Bilik D, Brown MB et al. Medical costs associated with type 2 diabetes complications and comorbidities. *Am J Manag Care* 2013;19(5):421-430.

(9) Siu AL. Screening for Abnormal Blood Glucose and Type 2 Diabetes Mellitus: U.S. Preventive Services Task Force Recommendation Statement. *Ann Intern Med* 2015;163(11):861-868.

(10) Dall TM, Narayan KM, Gillespie KB et al. Detecting type 2 diabetes and prediabetes among asymptomatic adults in the United States: modeling American Diabetes Association versus US Preventive Services Task Force diabetes screening guidelines. *Popul Health Metr* 2014;12:12.

(11) Dybicz SB, Thompson S, Molotsky S, Stuart B. Prevalence of diabetes and the burden of comorbid conditions among elderly nursing home residents. *Am J Geriatr Pharmacother* 2011;9(4):212-223.

(12) Congressional Budget Office. Insurance Coverage Provisions of the Affordable Care Act-CBO's April 2014 Baseline. *CBO* 2014;Available at: URL: https://www.cbo.gov/sites/default/files/cbofiles/attachments/43900-2014-04-ACAtables2.pdf.

(13) American Diabetes Association. Fast Facts: Data and Statistics about Diabetes. *ADA* 2015 December 1;Available at: URL: http://professional2.diabetes.org/admin/UserFiles/0%20-%20Sean/Documents/Fast_Facts_12-2015a.pdf.

(14) Centers for Disease Control and Prevention. Age-Adjusted Percentage of Adults with Diabetes Using Diabetes Medication, by Type of Medication, United States, 1997-2011. *CDC* 2013 November 19;Available at: URL: http://www.cdc.gov/diabetes/statistics/meduse/fig2.htm.

(15) Courtemanche T, Mansueto G, Hodach R, Handmaker K. Population health approach for diabetic patients with poor A1C control. *Am J Manag Care* 2013;19(6):465-472.

(16) Zhang X, Geiss LS, Cheng YJ, Beckles GL, Gregg EW, Kahn HS. The missed patient with diabetes: how access to health care affects the detection of diabetes. *Diabetes care* 2008;31(9):1748-1753.

(17) Zhang X, Bullard KM, Gregg EW et al. Access to health care and control of ABCs of diabetes. *Diabetes care* 2012;35(7):1566-1571.

(18) Hogan DR, Danaei G, Ezzati M, Clarke PM, Jha AK, Salomon JA. Estimating The Potential Impact Of Insurance Expansion On Undiagnosed And Uncontrolled Chronic Conditions. *Health Aff (Millwood )* 2015;34(9):1554-1562.
